# Substitution of CH_3_ by CH_2_F in 2‐Methylerythritol Cyclodiphosphate Triggers Potent Inhibition of IspG with Concomitant Fluoride Ion Expulsion

**DOI:** 10.1002/chem.202502471

**Published:** 2025-10-22

**Authors:** Clea Witjaksono, Vivien Herrscher, Hannah Jobelius, Nathan Noël, Fabien Massicot, Jean‐Luc Vasse, Jean‐Bernard Behr, Myriam Seemann

**Affiliations:** ^1^ Equipe Chimie Biologique et Applications Thérapeutiques Institut de Chimie de Strasbourg UMR 7177 Université de Strasbourg/CNRS 4, rue Blaise Pascal Strasbourg 67070 France; ^2^ Université de Reims Champagne‐Ardenne CNRS ICMR Reims 51100 France

**Keywords:** inhibitors, IspG (GcpE), MEP pathway, metalloenzyme, suicide substrate

## Abstract

IspG (also known as GcpE) is a key [4Fe‐4S] metalloenzyme that catalyzes the penultimate step of the methylerythritol phosphate (MEP) pathway, a well‐established target for the development of new antimicrobials. This oxygen‐sensitive enzyme mediates the reductive dehydroxylation of 2‐*C*‐methyl‐d‐erythritol 2,4‐cyclodiphosphate (MEcPP) to (*E*)‐4‐hydroxy‐3‐methylbut‐2‐en‐1‐yl diphosphate (HMBPP), requiring electron transfer proteins to deliver two electrons needed for catalysis. To probe the mechanism of IspG and access new mechanism‐based inhibitors, we synthesized a substrate analogue, monofluoromethyl‐d‐erythritol cyclodiphosphate, in which the natural methyl group of MEcPP is replaced by a CH_2_F group. This analogue proved to be a potent inhibitor of IspG. This study also demonstrates that electron capture is a prerequisite for inhibition and that the inhibitor leads to fluoride release in the IspG‐catalyzed reaction. Together, these results provide further support for the involvement of a carbanionic intermediate in the IspG mechanism.

## Graphical abstract

The fluoromethyl analogue of MEcPP was identified as a very potent inhibitor of the oxygen‐sensitive [4Fe‐4S] metalloenzyme IspG. In the course of the IspG‐catalyzed transformation, this novel compound undergoes fluoride elimination, offering additional evidence for the involvement of a carbanionic intermediate in the mechanism of this enzyme.

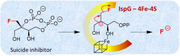

## 1 Introduction

Antibiotics have revolutionized modern medicine by allowing the effective treatment of a myriad of bacterial infections. However, the emergence of antimicrobial resistance (AMR) threatens these advancements, posing a major public health challenge.^[^ ^1^, ^2^, ^3^, ^4^ ^]^ One promising approach to address this crisis is the search for new therapeutic targets for anti‐infective drug development.^[^ ^1^, ^2^, ^3^, ^4^ ^]^ In this context, the methylerythritol phosphate (MEP) pathway affording ultimately isopentenyl diphosphate (IPP) and dimethylallyl diphosphate (DMAPP), the two building blocks essential for the biosynthesis of all isoprenoids, is a promising route. Indeed, in many pathogenic bacteria and in apicomplexan parasites, the production of these precursors is mediated through the MEP pathway, which is absent in humans, making it an attractive target for drug discovery.^[^ ^5^, ^6^ ^]^

**Figure 1:**
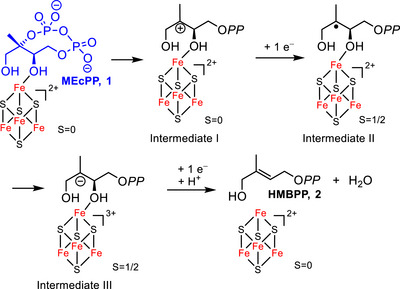
Proposed mechanism for IspG.

The MEP pathway consists of seven enzymatic steps (see Figure S1 in the Supplementary Information) that have been the subject of several investigations with the ultimate goal to identify new drugs with unprecedented modes of action.^[^ ^5^, ^6^, ^7^, ^8^, ^9^ ^]^ Among these transformations, the last two are catalyzed by IspG and IspH, two metalloenzymes harboring an oxygen‐sensitive [4Fe‐4S]^2+^ cluster essential for catalysis, rendering their investigations delicate and leaving them highly underexplored.^[^ ^10^, ^11^, ^12^, ^13^, ^14^ ^]^ Here, we focus our efforts on IspG (also known as GcpE), which catalyzes the penultimate step of the MEP pathway, converting 2‐*C*‐methyl‐d‐erythritol 2,4‐cyclodiphosphate (MEcPP, **1**) into (*E*)‐4‐hydroxy‐3‐methylbut‐2‐en‐1‐yl diphosphate **2** (HMBPP, Figure 1).^[^ ^10^, ^11^, ^12^, ^13^, ^15^, ^16^, ^17^, ^18^, ^19^ ^]^

The mechanism catalyzed by IspG is a reductive dehydroxylation that involves unprecedented bioinorganic and bioorganometallic intermediates. It was proposed that after binding of MEcPP via its 3‐OH to the [4Fe‐4S]^2+^ cluster of IspG, the cyclodiphosphate ester leaves the C‐2 position to form a carbocationic intermediate that further evolves toward a carbanion via electron transfers, followed by subsequent 3‐OH/H_2_O elimination to afford HMBPP.^[^ ^12^, ^20^, ^21^, ^22^, ^23^, ^24^, ^25^ ^]^ While many aspects of this mechanism have been clarified using enzymology and isotopic exchange, EPR/ENDOR spectroscopy, and X‐ray crystallography, certain intermediates, including possible radical or carbanion species, remain to be fully characterized.^[^ ^20^, ^21^, ^22^, ^23^, ^24^, ^25^ ^]^ Understanding the subtleties of this enzymatic process, notably with the aid of fine‐tuned substrate analogues,^[^ ^26^, ^27^ ^]^ could offer new insights for the development of inhibitors targeting IspG, potentially leading to new anti‐infectives to combat multidrug‐resistant bacteria and resistant apicomplexan such as *Plasmodium falciparum* responsible for malaria.

**Scheme 1:**
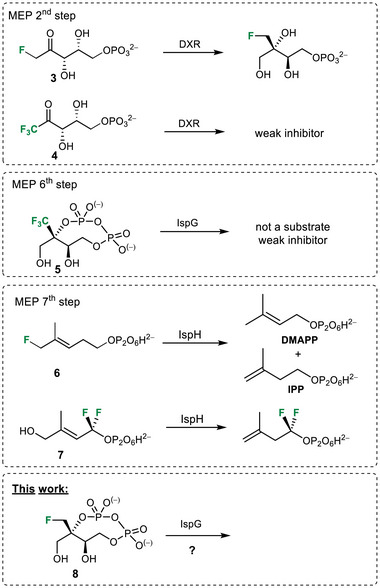
Fluorinated artificial substrates (H→F) processed by the enzymes DXR, IspG, and IspH in the MEP pathway.

Besides, structural modification of protein‐targeting ligands by incorporation of fluorinated functionalities is an important stratagem in medicinal chemistry and drug discovery.^[^ ^28^, ^29^, ^30^, ^31^ ^]^ The van der Waals radius of ^19^F (1.47 Å) and the C‐F bond length (1.41 Å) lie between those of hydrogen (radius = 1.20 Å and C‐H = 1.09 Å) and oxygen (radius = 1.52 Å and C‐O = 1.43 Å), so that C‐F is considered bioisosteric of C‐H or C‐OH.^[^ ^32^ ^]^ Since fluorine is a small atom, the lightest member of the halogens, insertion of a single fluorine into the structure of a biologically active compound causes weak steric perturbation. The main objectives of fluorine insertion are commonly summed up with the acronym FABS (Fluorine Atom for Biochemical Screening) and range from ^19^F‐NMR analyses of biological events,^[^ ^33^ ^]^ to the design of F‐containing analogues of biologically important molecules as radiopharmaceuticals^[^ ^34^ ^]^ or enzyme inhibitors.^[^ ^35^, ^36^ ^]^ In this latter case, the high electronegativity of fluorine and its leaving group ability may result in unusual metabolic pathways ending in enzyme inactivation. The monofluoromethyl trigger has mainly been exploited in the design of mechanism‐based inhibitors of enzymes that generate an electron‐rich intermediate (an α‐carbanion equivalent) able to expel fluoride.^[^ ^37^, ^38^, ^39^, ^40^, ^41^, ^42^ ^]^ As regards the MEP pathway, a couple of fluorinated analogues have been prepared and assayed. Some of them, such as **3–7** (Scheme 1), are exact structural copies of enzymes’ substrates/products, which integrate F in place of H.^[^ ^43^, ^44^, ^45^, ^46^, ^47^, ^48^ ^]^ Interestingly, the mono‐fluorinated artificial substrates are processed by their corresponding enzymes, although at a lower rate.

Combining these preliminary observations, namely, the ability of monofluorinated analogues to act as substrates and their capacity to undergo fluoride elimination from a carbanionic intermediate, we designed monofluoromethyl‐d‐erythritol cyclodiphosphate **8** as a new possible mechanism‐based inhibitor of IspG. The synthesis of **8** and its evaluation as a substrate/inhibitor are described herein.

## 2 Results and Discussion

C1‐fluorinated building block insertion is a well‐established methodology when applied to trifluoro‐ or difluoromethyl‐containing synthetic targets.^[^ ^49^, ^50^, ^51^, ^52^, ^53^, ^54^ ^]^ On the contrary, due to the lack of effective CH_2_F donors, direct monofluoromethylation strategies are rather underdeveloped, so that alternative tactics must be considered in this case. Functional group interconversions (OH → F, for instance) are particularly primed, and a number of reagents are available to succeed in the conversion of alcohols into fluorinated analogues.^[^ ^55^, ^56^ ^]^

Our strategy for the synthesis of fluoromethyl‐d‐erythritol cyclodiphosphate **8** relied on such a reaction applied to alcohol **9** (Table 1), which was prepared in 5 steps from diacetone‐d‐glucose after improvement and clarification of existing procedures (see the Supporting Information). Indeed, the pivotal quaternary C‐2 center in the structure of **8** could be generated from **9** by deoxofluorination, and the stereogenic C‐3(OH) in **8** could originate from C‐3(OH) of **9**.

**Table 1:** Deoxofluorination of primary alcohol **9**.

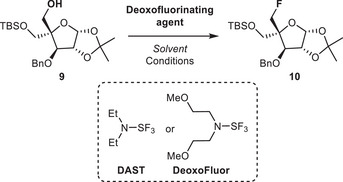				
Entry	Reagent	Solvent	Conditions	Yield ^[^ ^a^ ^]^
1	DAST (2 equiv)	CH_2_Cl_2_	−20 °C to rt, 4 hours	6%
2	DAST (2 equiv)	CH_2_Cl_2_	0 °C, 2 hours	29%
3	DAST (2 equiv)	PhMe	60 °C, 2 hours	35%
4	DAST (3 equiv)	PhMe	60 °C, 2 hours	45%
5	DAST (3 equiv)	PhMe	MW 150 W, 20 minutes	65%
6	DeoxoFluor (1.2 equiv)	CH_2_Cl_2_	−20 °C to rt, 8 hours	13%
7	DeoxoFluor (1.5 equiv)	CH_2_Cl_2_	−78 °C to rt, 4 hours	28%
8	DeoxoFluor (3 equiv)	PhMe	MW 150 W, 20 minutes	0%

^[a]^ isolated yield after purification of **10**.

Deoxofluorination of **9** was studied first. Following typical protocol applied to primary alcohols,^[^ ^57^ ^]^ **9** was treated with diethylaminosulfur trifluoride (DAST, 2 equiv) at low temperature for 4 hours, to afford the expected fluoride **10** in very low yield (Table 1, entry 1). An increase in yield was observed when the temperature was stepped up, culminating at 45% at 60 °C with 3 equiv of DAST (Table 1, entries 2–4). To our delight, microwave activation proved more efficient, giving **10** in 65% isolated yield. Some attempts were conducted with DeoxoFluor to improve the yields, but to no avail (Table 1, entries 6–8).

**Scheme 2:**
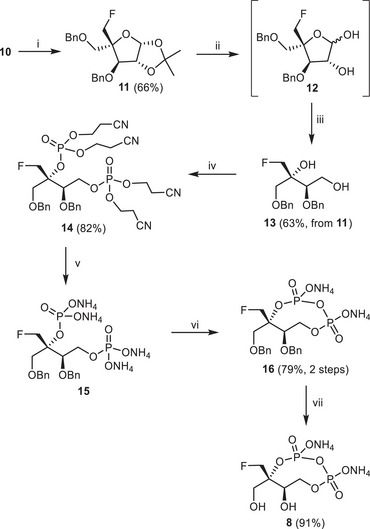
Synthesis of compound **8**. Reagents and conditions, i) TBAF (1.2 equiv), then NaH (2 equiv), BnBr (1.5 equiv) (66%, 2 steps); ii) AcOH/water/THF (8/1/1), 80 °C, 6 hours; iii) NaIO_4_ (2 equiv), then NaBH_4_ (2 equiv), in MeOH/water (63%, 3 steps); iv) *Ph*‐tetrazole (6 equiv), bis(2‐cyanoethyl)‐N,N‐diisopropylphosphoramidite (3 equiv), DCM, rt, then H_2_O_2_ (10 equiv), rt; (82%) v) NH_4_OH 30%/MeOH (3/1), 55 °C, 24 hours, then ion exchange resin (NH_4_^+^ form); vi) DCC (6 equiv), DMF, rt, 5 hours, (79%); vii) H_2_, Pd/C, MeOH, rt, 18 hours, then ion exchange resin (NH_4_^+^ form), (91%).

Then, the transformation of **10** to key intermediate **13**, suitable for bis‐phosphorylation, began with a protecting‐group interconversion at C‐4 from − CH_2_OTBS to − CH_2_OBn under standard conditions (deprotection with tetrabutylammonium fluoride, followed by benzylation with sodium hydride/benzyl bromide, 66% overall yield) to afford **11** as shown in Scheme 2. The 2‐*C*‐fluoromethyl‐d‐erythritol core was obtained after deprotection of 1,2‐isopropylidene under acidic conditions followed by standard periodate oxidation/sodium borohydride reduction, yielding **13** in 63% overall yield from **11**. Bis‐phosphorylation of hydroxyls at C‐2 and C‐4 was performed with an excess of bis(2‐cyanoethyl)‐*N*,*N*‐diisopropylphosphoramidite (3 equiv) and subsequent oxidation with hydrogen peroxide to afford bis‐cyanoethyl bis‐phosphate **14** in 82% yield. Cyanoethyl protecting groups were smoothly removed by treatment with aqueous ammonia in methanol. After 24 hours at 55 °C, the resulting solution was concentrated and passed through a column of ion‐exchange resin (Dowex 50WX‐8, NH_4_^+^ form), which retained coproducts resulting from degradation of the generated acrylonitrile and afforded bis‐phosphate **15** as its ammonium salt in almost pure form.

After lyophilization, **15** was solubilized in DMF and treated with DCC to elicit internal phosphate coupling. The expected cyclic pyrophosphate **16** was obtained in 79% yield after purification by reversed‐phase chromatography using water as eluent. A characteristic ^2^ *J*_P‐P_ coupling constant of 23 Hz was observed in the ^31^P{1H}‐NMR spectrum of **16**, consistent with the new P‐O‐P sub‐structure. Finally, hydrogenolysis of the OBn groups under an H_2_ atmosphere in the presence of Pd on activated charcoal (10%, on a Pd basis) followed by ion exchange chromatography on DOWEX 50WX8 resin (NH_4_^+^ form) and purification by reversed‐phase chromatography using water as an eluent afforded **8**, the fluoromethyl analogue of MEcPP.

Next, we evaluated the ability of **8** to act as an IspG inhibitor. *E. coli* IspG contains an oxygen‐sensitive [4Fe‐4S]^2+^ center coordinated by three conserved cysteines and, likely, a glutamate, as suggested by Mössbauer spectroscopy data on *E. coli* IspG and crystal structures of homologues from *Aquifex aeolicus* and *Thermus thermophilus*.^[^ ^58^, ^59^, ^60^ ^]^ The role of this [4Fe‐4S] cluster is triple as it i) is involved in substrate recognition by supplanting its noncysteine ligand by the 3‐OH of the substrate, ii) mediates electron transfer, and iii) promotes the 3‐OH elimination with its apical iron playing the role of a Lewis acid.^[^ ^10^, ^11^, ^12^, ^13^ ^]^

**Figure 2:**
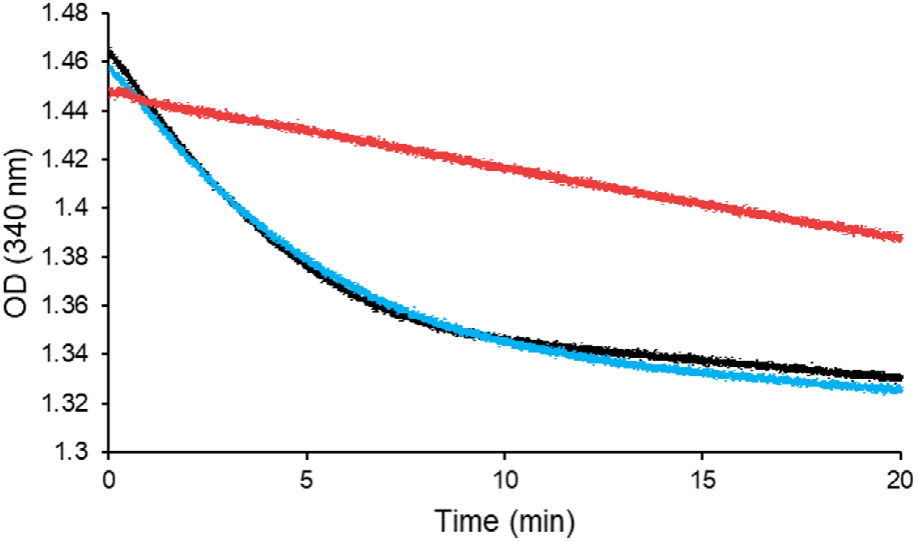
IspG enzymatic assays: decrease in the NADPH absorbance at 340 nm with and without **8**.

*E. coli* IspG was produced and purified under a strictly inert (N_2_) atmosphere following our recently described protocol,^[^ ^27^ ^]^ which enables isolation of pure samples of the active dimeric form.^[^ ^61^ ^]^ Since IspG catalyzes a reductive dehydroxylation, it needs to be coupled to a reduction system. In *E. coli*, the natural flavodoxin (FldA)/flavodoxin reductase (FpR1)/NADPH system is essential for delivering electrons to IspG, enabling its catalytic activity in vivo.^[^ ^62^ ^]^ Consequently, *E. coli* IspG activity in vitro was assessed under anaerobic conditions by monitoring the NADPH consumption in the presence of optimized concentrations of its redox partners.^[^ ^27^ ^]^ Under these conditions, the activity of *E. coli* IspG was around 200 nmol min^−1^ mg^−1^ (Figure 2, black curve). In a further series of experiments, we checked the capacity of monofluoromethyl‐d‐erythritol cyclodiphosphate **8** to disrupt the course of the natural enzymatic process. Incubating **8** (250 µM) with equimolar MEcPP (250 µM), *E. coli* IspG, and the reduction system led to a very minor drop in IspG activity (11%) that decreased from 200 nmol min^−1^ mg^−1^ to 177 nmol min^−1^ mg^−1^ when the test was initiated by the addition of IspG (Figure 2, blue curve). However, when *E. coli* IspG was first pre‐incubated for 15 minutes at 37 °C in the presence of **8** (250 µM) and the reduction system, followed by the late addition of MEcPP (250 µM) to start the enzymatic reaction, the resulting *E. coli* IspG activity dropped to 21 nmol min^−1^ mg^−1^, revealing an inhibition of 89% (Figure 2, red curve). Subsequently, *E. coli* IspG was again first pre‐incubated for 15 minutes at 37 °C in the presence of **8** (250 µM) but omitting the reduction system. This mixture was thereafter added to a solution of MEcPP (250 µM) and the reduction system to initiate the reaction. Interestingly, the obtained progress curve (Figure S2, green) was similar to the progress curve of the IspG assay in the absence of **8**, revealing no IspG inhibition (Figure S2, black). These experiments demonstrate that the NADPH/flavodoxin reductase/flavodoxin system is essential during pre‐incubation for effective IspG inhibition, suggesting that **8** might enter the first steps of the enzymatic reaction, at least until the first electron transfer, to promote inhibition.

Conditions: NADPH (2 mM), FldA (60 µM), FpR1 (60 µM), IspG (4 µM), MEcPP (250 µM), and **8** 0 µM (black) or 250 µM (blue and red) in 50 mM Tris·HCl buffer pH 8 at 37 °C. The reaction was initiated by the addition of IspG (black and blue). MEcPP was added after the 15 minutes pre‐incubation of IspG in the presence of **8** at 37 °C (red). The final volume of the assay was 150 µL. The time interval chosen to determine the IspG activity was chosen with respect to a maximum of 20% substrate conversion.

**Figure 3:**
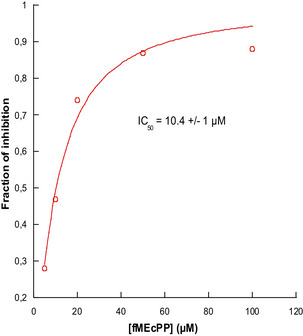
Fraction of inhibition of IspG versus concentration of **8**.

To compare the inhibition potency of **8** with that of 2‐*C*‐vinyl‐d‐erythritol cyclodiphosphate (VEcPP), a 2‐vinyl analogue of MEcPP that is a very potent IspG inhibitor requiring also electron transfer to mediate IspG inhibition,^[^ ^26^ ^]^ further assays were conducted to determine the IC_50_ value of **8**. Therefore, IspG was preincubated with varying concentrations of **8** for 30 minutes in the presence of the reduction system prior to the addition of MEcPP (250 µM) to initiate the catalysis. Kinetic data led to an apparent IC_50_ value of 10.4 ± 1 µM (Figures 3 and S3). When tested under the same conditions, the apparent IC_50_ value obtained for VEcPP was 19.5 ± 1.3 µM (Figure S4) highlighting the high inhibition potency of **8**. It should be mentioned that the previously reported IC_50_ value of VEcPP (62.7 ± 2.1 µM was obtained under different conditions and using an IspG preparation that was not exclusively in its active dimeric form.^[^ ^26^ ^]^

Control experiments similar to those reported for VecPP^[^ ^26^ ^]^ were further carried out to confirm that **8** targets IspG and not the reduction system. The results (Figure S5) indicate that **8** does not interfere with the reduction system.

**Scheme 3:**
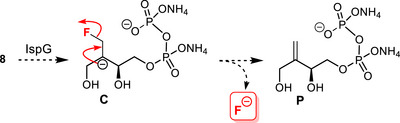
Expected processing of **8** by IspG via anionic species **C**, with release of fluoride and alkene **P**.

As **8** is a substrate analogue, it might enter the active site and bind to the [4Fe‐4S]^2+^ cluster via its C3‐OH, similarly to MEcPP (Figure 1). If ring opening occurs, the consecutive transfer of two electrons by the reduction system would generate carbanion **C**, able to undergo fluoride elimination to yield product **P** (Scheme 3).

**Figure 4:**
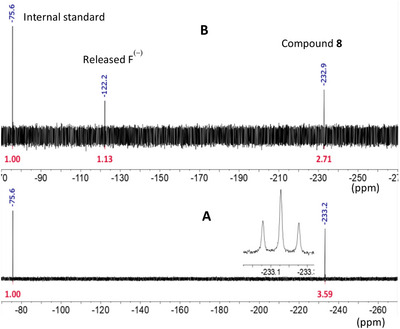
Comparison of ^19^F NMR spectra of starting material **A**) and crude enzymatic reaction mixture **B**), in the presence of CF_3_COONH_4_ as an internal standard. Chemical shifts are quoted in blue, and integrations in red.

To test this hypothesis, an incubation of IspG was performed in the presence of the reduction system by replacing the MEcPP substrate with a sample of **8** (which contained *ca* 10 mol% of ammonium trifluoromethyl acetate as an internal standard) for 16 hours at pH 8 under anaerobic conditions. The volume of the assay was increased (500 µL instead of 150 µL), and a higher concentration of IspG (30 µM instead of 4 µM) was used in these experiments to secure quantifiable conversion of **8**. Before removing the sample from the glove box, the enzymes were eliminated to prevent any loss of paramagnetic iron ions that would occur upon exposure of IspG to oxygen. The resulting solution was lyophilized and resuspended in D_2_O before being analyzed by ^19^F‐NMR. Interestingly, whereas ^1^H‐NMR signals of the reaction mixtures were difficult to assign (see the Supporting Information) due to the presence of multiple components,^[^ ^63^ ^]^ a stringent analysis could result from the very clean ^19^F‐NMR spectrum (Figure 4). Indeed, only the remaining **8** (−233 ppm), the internal standard (−76 ppm) and a new singlet at −122 ppm were apparent after enzyme‐mediated transformation. Relative integrations clearly demonstrate that the disappearance of fluoromethyerythritol cyclodiphosphate **8** occurred in favor of the new signal at −122 ppm, a chemical shift in agreement with free fluoride ion as described by others in similar experiments.^[^ ^64^ ^]^ Quantification of the process by relative integrations toward CF_3_COONH_4_ rules out possible contamination of the reaction mixture by exogeneous fluoride ions, such as those arising from glassware or NMR tubes.^[^ ^64^ ^]^ In order to verify that the internal standard does not interfere with the IspG‐catalyzed reaction, an IspG assay was carried out in the presence of CF_3_COOH (TFA). The results show that a TFA concentration of 250 µM, which is much higher than the concentration of the internal standard, has no impact on IspG activity (Figure S6).

## 3 Conclusion

In conclusion, we have successfully synthesized a novel substrate analogue of MEcPP, a key component of the MEP pathway. This new analogue **8**, in which the native methyl group is replaced by a monofluoromethyl moiety, was synthesized via a multi‐step synthetic approach starting from diacetone‐d‐glucose. Biological evaluation revealed that **8** is a highly potent inhibitor of *E. coli* IspG. Notably, its inhibitory activity depends on electron capture to convert it into the species responsible for enzyme inhibition, categorizing **8** as a suicide substrate. The ability of **8** to enter the IspG active site and release fluoride provides further evidence for the involvement of the carbanionic intermediate **III** in the IspG‐catalyzed mechanism.

## Supporting Information

The authors have cited additional references within the Supporting Information.^[^ ^65^, ^66^, ^67^, ^68^, ^69^ ^]^ Supporting Information includes experimental procedures (chemistry and biology), analytical characterization and NMR spectra for compounds **8**–**16**. ^1^H and ^19^F NMR spectra of the crude enzymatic reaction mixture are also included.

## Conflict of Interest

The authors declare no conflict of interest.

## Supporting information



Supporting Information

## Data Availability

The data that support the findings of this study are available in the supplementary material of this article.
