# Supplementary material for: Substitution of CH3 by CH2F in 2‐Methylerythritol Cyclodiphosphate Triggers Potent Inhibition of IspG with Concomitant Fluoride Ion Expulsion
Source: Chemistry. 2025 Oct 22;31(66):e02471. doi: 10.1002/chem.202502471 (PMC12648460; doi:10.1002/chem.202502471)
Supplement: Supplementary file 1 — Supporting Information [file CHEM-31-e02471-s001.pdf]

Substitution of CH_3 by CH_2F in 2-Methylerythritol Cyclodiphosphate Triggers Potent Inhibition of IspG with Concomitant Fluoride Ion Expulsion

Clea Witjaksono,[a] Vivien Herrscher,[b] Hannah Jobelius,[a] Nathan Noël,[b] Fabien Massicot,[b] Jean-Luc Vasse [b] Jean-Bernard Behr*[b] and Myriam Seemann*[a]

[a] Equipe Chimie Biologique et Applications Thérapeutiques, Institut de Chimie de Strasbourg, UMR 7177, Université de Strasbourg/CNRS, 4, rue Blaise Pascal, 67070 Strasbourg, France
E-mail: mseemann@unistra.fr

[b] Dr. V. Herrscher, N. Noël, Dr. F. Massicot, Dr. J.-L. Vasse, Prof. Dr. J.-B. Behr
Université de Reims Champagne-Ardenne, CNRS, ICMR, Reims, France
E-mail: jb.behr@univ-reims.fr

Table of Contents

Figure S1, description of the MEP pathway p S2

Chemistry

General methods p S3

Synthesis of compound 9, NMR spectra (^1H, ^{13}C and NOESY) of 9 p S4

Experimental procedures for the synthesis of 10-16 and 8 p S7

NMR spectra of compounds 10-16 and 8 p S12

Biology

Biological procedures p S27

Figure S2 and S3 p S28

Figure S4 and S5 p S29

Figure S6 p S30

^1H and ^{19}F-NMR spectra of enzymatic mixtures p S31

References p S32

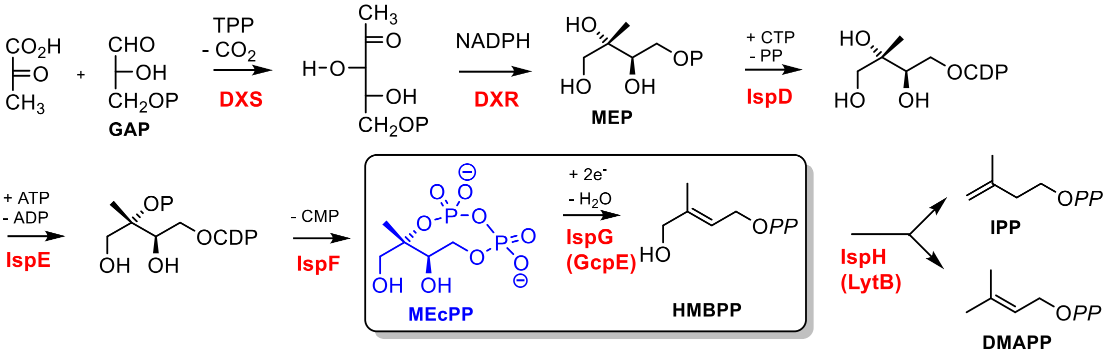

Figure S1: Methylerythritol phosphate (MEP) pathway. The step catalyzed by IspG (also called GcpE) is drawn with a border.

Chemistry

General methods

Reactants and reagents were purchased from standard suppliers (Sigma-Aldrich, Alfa-Aesar, Fisher Scientific) and were used without further purification. Methanol, dichloromethane, diethyl ether, toluene and tetrahydrofuran were dried on an Innovative Technology, Inc. Pure Solv MD system; other solvents were dried according to conventional methods. Reactions were monitored by TLC using Silica gel F254 (0.2 mm) TLC plates, detection being carried out by spraying with an aqueous solution of $KMnO_4$ (2%) / Na_2CO_3 (4%) or alcoholic solutions of phosphomolybdic acid or *p*-anisaldehyde, followed by heating. Column chromatography purifications were performed over silica gel M 9385 (40–63 µm) Kieselgel 60. NMR spectra were recorded on Bruker AC 250 (250 MHz for ^{1}H, 63 MHz for ^{13}C and 101 MHz for ^{31}P), 500 (500 MHz for ^{1}H, 126 MHz for ^{13}C and 202 MHz for ^{31}P) or 600 (600 MHz for ^{1}H, 150 MHz for ^{13}C and 202 MHz for ^{31}P) spectrometers. Chemical shifts are expressed in parts per million (ppm) and were calibrated to deuterated or residual non-deuterated solvent peaks for ^{1}H and ^{13}C spectra. Coupling constants are in Hz and splitting pattern abbreviations are: br, broad; s, singlet; d, doublet; t, triplet; m, multiplet. DEPT and JMOD 1D NMR experiments, COSY, HSQC, and HMBC 2D NMR experiments were used to confirm the NMR peak assignments for all compounds. Optical rotations were determined at 20 °C (unless otherwise stated) with a Perkin-Elmer Model 241 polarimeter. High Resolution Mass Spectra (HRMS) were performed on Q-TOF Micro micromass positive ESI (CV = 30 V).

Synthesis of compound 9, ^{1}H, ^{13}C, and NOESY NMR spectra of 9.

The synthesis of key 4-hydroxymethyl-1,2-O-isopropylidene-3-O-benzyl-α-D-xylofuranose **A** (Scheme S1) has already been reported in the literature, in four steps from 1,2;5,6-di-O-isopropylidene-α-D-glucofuranose without purification of intermediates, using an aldol-crossed Cannizzaro reduction sequence as critical reaction.[1] These procedures were followed, without modifications.

Diastereoselective protection of hydroxymethyl groups in **A** has also been devised, though with contrasting results. According to the literature, silylation of **A** could afford **9** [2] or its diastereoisomer **9'**,[3] depending on the temperature. More surprisingly, the given NMR data for **9** and **9'** were strictly identical. Due to these uncertainties, we settled our own reaction conditions. In our hands, the treatment of **A** with TBSCl in the presence of dibutyltin oxide triggers selective beta-protection to afford **9** (61% yield), the configuration of which was unambiguously confirmed by nOe NMR experiments. The only by-product observed here was the bis-silylated homologue **9''**, isolated in 19% yield. Analytical data are in agreement with those from reference [2].

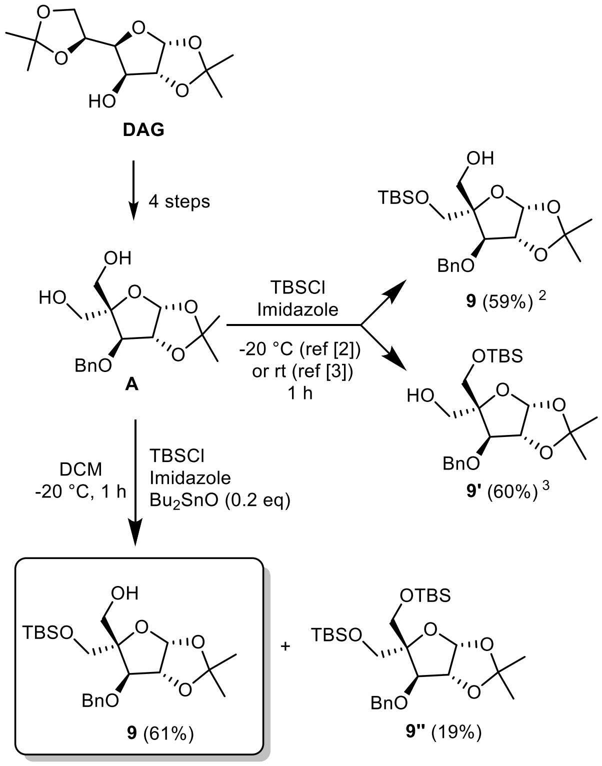

Scheme S1. Synthesis of **9** from diacetone-D-glucose.

Experimental procedure for the synthesis of **9**:

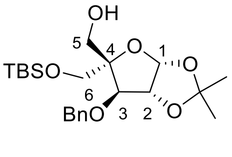

Under argon atmosphere, to a solution of compound **A** (130 mg, 0.31 mmol, 1 equiv) in dry dichloromethane (5 mL) at -20 °C were added sequentially imidazole (63 mg, 0.92 mmol, 3 equiv),

dibutyltin oxide (30 mg, 0.06 mmol, 0.2 equiv) and *t*-butyldimethylsilyl chloride (46 mg, 0.31 mmol, 1 equiv). The mixture was stirred for 1 h at -20 °C. Then, it was warmed to room temperature and washed with water (3x10 mL). The combined aqueous layers were extracted with dichloromethane (10 mL). The combined organic layers were dried over $MgSO_4$, filtrated and concentrated. The crude was purified by silica gel chromatography (petroleum ether/ethyl acetate, 9/1) to afford **9** as white crystals (79 mg, 0.19 mmol, 61%).

^{1}H NMR (500 MHz, Chloroform-*d*) δ 7.39 – 7.28 (m, 5H, Ph), 6.01 (d, $J^3_{H\text{-}H}$= 4.3 Hz, 1H, H^1), 4.75 – 4.71 (m, 2H, H^2, Bn), 4.57 (d, $J^2_{H\text{-}H}$= 11.6 Hz, 1H, Bn), 4.21 (d, $J^3_{H\text{-}H}$= 1.4 Hz, 1H, H_3), 3.91 (d, $J^2_{H\text{-}H}$= 12.0 Hz, 1H, H^5a), 3.70 (s, 2H, H^6), 3.65 (d, $J^2_{H\text{-}H}$= 12.0 Hz, 1H, H^5b), 1.55 (s, 3H, CH_3), 1.35 (s, 3H, CH_3), 0.86 (s, 9H, Si-*t*Bu), 0.04 (s, 3H, Si-CH_3), 0.03 (s, 3H, Si-CH_3). **^{13}C NMR (126 MHz, Chloroform-*d*)** δ 137.2 (Cq, Ph), 128.7 (Ph), 128.2 (Ph), 127.9 (Ph), 113.1 (Cq, ketal), 105.1 (CH, C^1), 89.6, 86.4, 85.2, 72.9, 64.7, 64.6, 27.5 (CH_3, ketal), 26.8 (CH_3, ketal), 26.0 (3xCH_3, Si-*t*Bu), 18.4 (Si-Cq), -5.3 (Si-CH_3), -5.4 (Si-CH_3).

<u>^{1}H-NMR</u> spectrum of 9 ($CDCl_3$, 500 MHz)

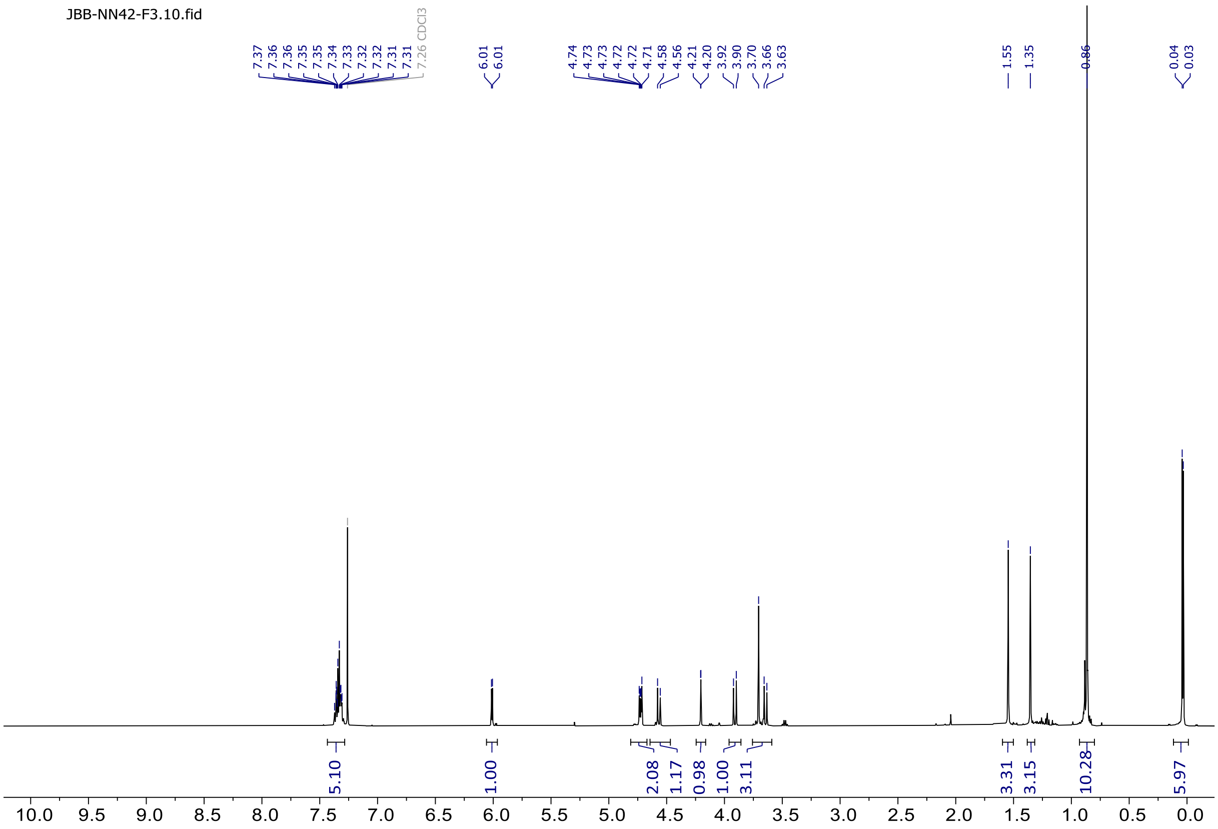

^{13}C-NMR spectrum of 9 ($CDCl_3$, 126 MHz)

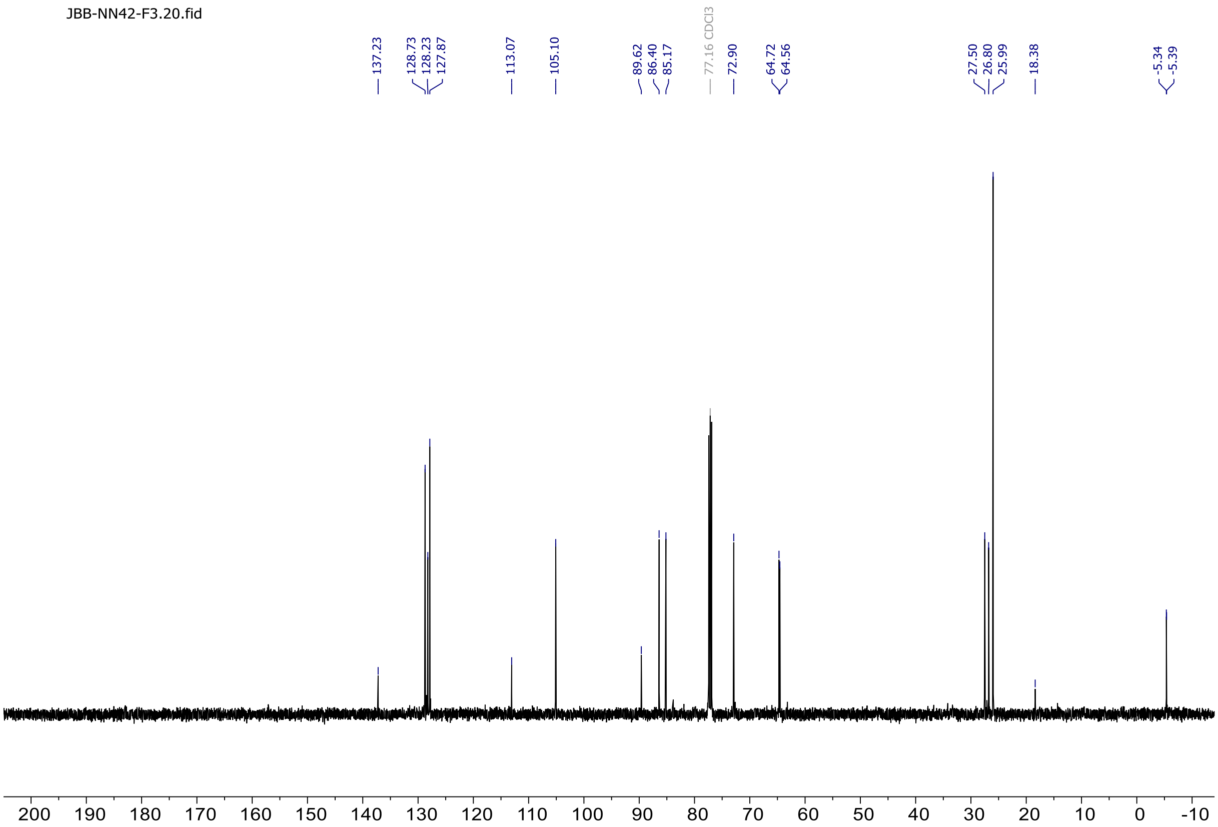

NOESY **spectrum of 9 ($CDCl_3$, 126 MHz)**

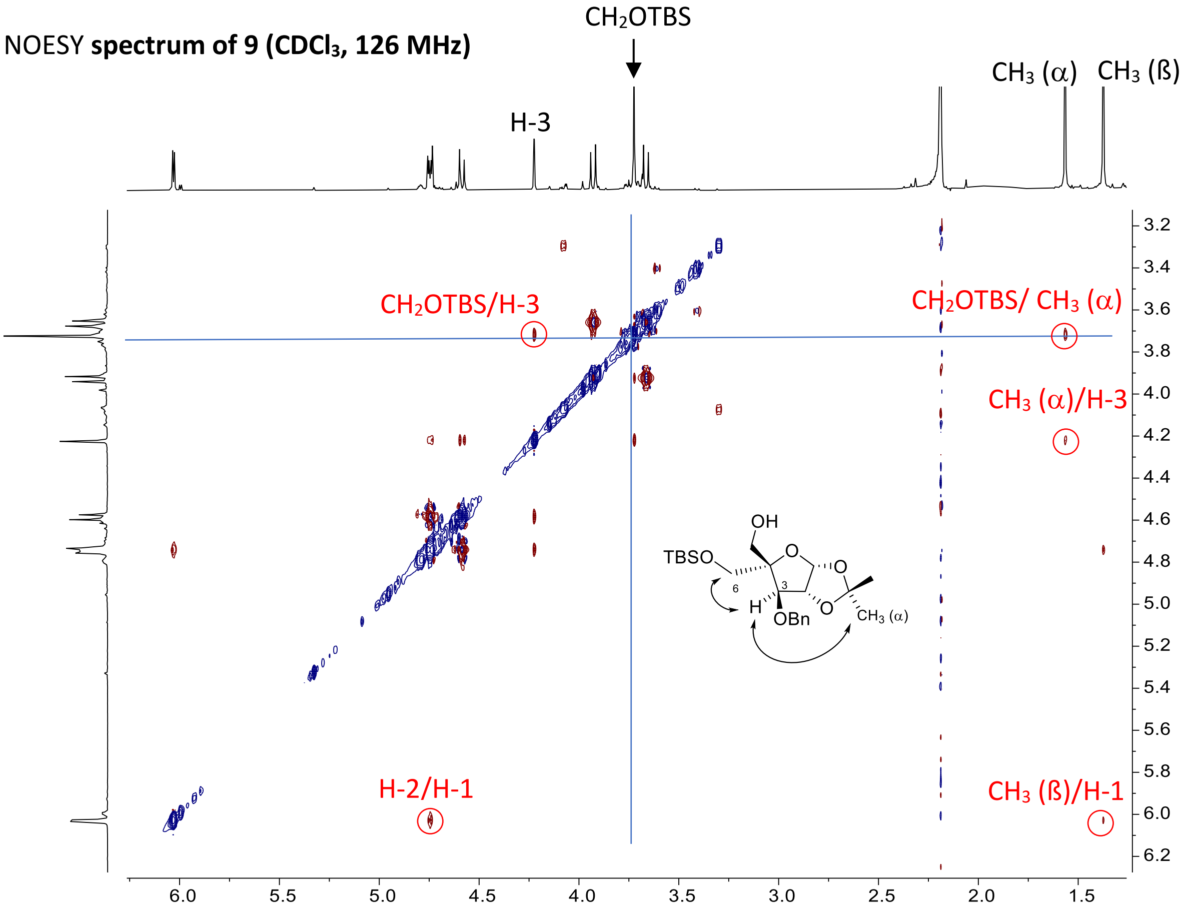

Experimental procedures for the synthesis of 10-16 and 8

3-*O*-benzyl-6-*O*-*t*-butyldimethylsilyl-5-fluoro-1,2-*O*-isopropylidene-β-L-arabinofuranose, **10**

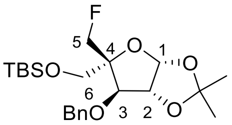

To a solution of DAST (110 µL, 0.83 mmol, 3 equiv) in dry toluene (1 mL) was added at 0 °C a solution of compound **9** (121 mg, 0.28 mmol, 1 equiv) in toluene (2 mL). The reaction mixture was submitted to microwave irradiation at 150 W for 20 min to reach a temperature between 120 and 160 °C. After being cooled to room temperature, the mixture was extracted with dichloromethane (4x5 mL) and concentrated. The crude residue was purified by flash column chromatography (petroleum ether/diethyl ether, 9/1) to afford **10** as a yellow oil (79 mg, 0.18 mmol, 65%).

^{1}H NMR (500 MHz, Chloroform-*d*) δ 7.47 – 7.28 (m, 5H, Ph), 5.99 (d, $J^3_{\text{H-H}}$= 4.3 Hz, 1H, H^1), 4.73 – 4.69 (m, 2H, Bn, H^2), 4.63 (dd, $J^2_{\text{H-F}}$= 25.2 Hz, $J^2_{\text{H-H}}$= 9.7 Hz, 1H, H^{5}a), 4.57 – 4.47 (m, 2H, H^{5}b, Bn), 4.16 (d, $J^3_{\text{H-H}}$= 1.4 Hz, 1H, H^3), 3.78 (d, $J^2_{\text{H-H}}$= 10.0 Hz, 1H, H^{6}a), 3.67 (dd, $J^2_{\text{H-H}}$= 10.0 Hz, $J^4_{\text{H-F}}$= 2.3 Hz, 1H, H^{6}b), 1.55 (s, 3H, CH$_3$), 1.35 (s, 3H, CH$_3$), 0.86 (s, 9H, *t*Bu), 0.04 (s, 3H, CH$_3$, Si), 0.03 (s, 3H, CH$_3$, Si). **^{13}C NMR (126 MHz, Chloroform-*d*)** δ 137.4 (Cq, Ph), 128.6 (Ph), 128.1 (Ph), 127.7 (Ph), 112.9 (Cq, ketal), 105.5 (CH, C^1), 88.8 (d, $J^2_{\text{C-F}}$= 16.3 Hz, Cq, C^4), 85.5 (CH, C^2), 84.1 (d, $J^3_{\text{C-F}}$= 3.4 Hz, CH, C^3), 83.2 (d, $J^1_{\text{C-F}}$= 171.2 Hz, CH$_2$, C^5), 72.4 (CH$_2$, Bn), 62.5 (d, $J^3_{\text{C-F}}$= 8.3 Hz, CH$_2$, C^6), 27.3 (CH$_3$, ketal), 26.6 (CH$_3$, ketal), 25.9 (CH$_3$, *t*Bu), 18.3 (Cq, *t*Bu), -5.4 (CH$_3$, Si), -5.5 (CH$_3$, Si). **^{19}F NMR (471 MHz, Chloroform-*d*)** δ -235.9 (t, $J^2_{\text{F-H}}$= 47.4 Hz). **IR (neat) σ** 2931, 4169, 1007, 836, 778. **HRMS (ESI$^+$) Calcd for $C_{22}H_{35}O_5NaSiF$ [M+Na]$^+$**= 449.2135; found= 449.2135. $[\alpha]^{25}_D$= -32.5° (c 1.0, CHCl$_3$).

3,6-di-*O*-benzyl-4-*C*-fluoromethyl-1,2-O-isopropylidene-β-L-arabinofuranose, **11**

Under argon atmosphere, to a solution of **10** (79 mg, 0.18 mmol, 1 equiv) in dry tetrahydrofuran (5 mL) at 0 °C was added tetrabutylammonium fluoride 1M (0.22 mmol, 220 µL, 1.2 equiv) and the mixture was stirred for 1 h at room temperature. The reaction mixture was then concentrated under reduced pressure. The crude residue was purified by flash column chromatography (petroleum ether/ethyl acetate, 6/4) to afford the intermediate **3-*O*-benzyl-4-*C*-(fluoromethyl)-1,2-*O*-isopropylidene-β-L-arabinofuranose** as a colorless oil (52 mg, 0.16 mmol, 89%).

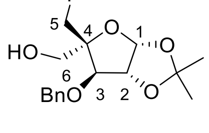

^{1}H NMR (500 MHz, Chloroform-*d*) δ 7.42 – 7.28 (m, 5H, Ph), 5.97 (dd, $J^3_{\text{H-H}}$= 4.5 Hz, $J^5_{\text{H-F}}$=1.5 Hz, 1H, H^1), 4.78 – 4.72 (m, 2H, H^2, Bn), 4.59 (dd, $J^2_{\text{H-F}}$= 36.6 Hz, $J^2_{\text{H-H}}$= 9.4 Hz, 1H, H^{5}a), 4.58 (s, 1H, Bn), 4.52 (dd, $J^2_{\text{H-F}}$= 37.3 Hz, $J^2_{\text{H-H}}$= 9.4 Hz, 1H, H^{5}b), 4.04 (d, $J^3_{\text{H-H}}$= 2.0 Hz, 1H, H^3), 3.72 (dd, $J^2_{\text{H-H}}$= 11.8 Hz, $J^4_{\text{H-F}}$= 2.1 Hz, 1H, H^{6}a), 3.67 (dd, $J^2_{\text{H-H}}$= 11.8 Hz, $J^4_{\text{H-F}}$= 1.5 Hz, 1H, H^{6}b), 1.55 (s, 3H, CH$_3$), 1.35 (s, 3H, CH$_3$). **^{13}C NMR (126 MHz, Chloroform-*d*)** δ 137.3 (Cq, Ph), 128.6 (Ph), 128.1 (Ph), 127.7 (Ph), 113.4 (Cq, ketal), 105.2 (d, $J^4_{\text{C-F}}$= 2.6 Hz, CH, C^1), 88.4 (d, $J^2_{\text{C-F}}$= 18.5 Hz, CH, C^4), 85.7 (CH, C^2), 83.6 (CH, C^3), 82.0 (d, $J^1_{\text{C-F}}$=

171.1 Hz, CH_2, C^5), 72.6 (CH_2, Bn), 62.0 (d, $J^{3}_{C\text{-}F}$= 7.1 Hz, CH_2, C^6), 27.3 (CH_3, ketal), 26.8 (CH_3, ketal). **^{19}F NMR (471 MHz, Chloroform-*d*)** δ -231.6, (t, $J^{2}_{F\text{-}H}$= 46.7 Hz). **IR (neat) σ** 3478, 2940, 1455, 1375, 1212, 1163, 1068, 698. **HRMS (ESI$^+$) Calcd for $C_{16}H_{21}O_5NaF$ [M+Na]$^+$**= 335.1271; found= 335.1271. $[\alpha]^{25}_{D}$= -35.2° (c 1.0, $CHCl_3$).

To a solution of **3-*O*-benzyl-4-*C*-(fluoromethyl)-1,2-*O*-isopropylidene-β-L-arabinofuranose** (213 mg, 0.68 mmol, 1 equiv) in dry dimethylformamide (10 mL) was then added NaH 60% (80 mg, 1.36 mmol, 2 equiv) and the mixture was stirred for 30 min. To the mixture at 0 °C was added benzyl bromide (0.12 mL, 1.02 mmol, 1.5 equiv). Then, it was warmed to room temperature for 4 h. Methanol (5 mL) was added for quenching and the mixture was concentrated under reduced pressure. Water (10 mL) was added and the aqueous layer was extracted with diethyl ether (2x10 mL). The combined organic layers were dried over $MgSO_4$, filtrated and concentrated. The crude was purified by silica gel flash column chromatography (petroleum ether/ethyl acetate, 95/5) to afford **11** as a slightly yellow oil (203 mg, 0.5 mmol, 74%).

^{1}H NMR (500 MHz, Chloroform-*d*) δ 7.36 – 7.26 (m, 10H, Ph), 5.98 (dd, $J^{3}_{H\text{-}H}$= 4.4 Hz, $J^{5}_{H\text{-}F}$= 1.5 Hz, 1H, H^1), 4.75 – 4.71 (m, 2H, Bn, H^2), 4.69 (dd, $J^{2}_{H\text{-}F}$= 32.5 Hz, $J^{2}_{H\text{-}H}$= 9.6 Hz, 1H, H^5a) 4.61 – 4.48 (m, 4H, H^5a, 3xBn), 4.12 (d, $J^{3}_{H\text{-}H}$= 1.8 Hz, 1H, H^3), 3.61 (dd, $J^{2}_{H\text{-}H}$= 9.8 Hz, $J^{4}_{H\text{-}F}$= 1.7 Hz, 1H, H^6a), 3.56 (dd, $J^{2}_{H\text{-}H}$= 9.8 Hz, $J^{4}_{H\text{-}F}$= 2.0 Hz, 1H, H^6b), 1.44 (s, 3H, CH_3), 1.34 (s, 3H, CH_3). **^{13}C NMR (126 MHz, Chloroform-*d*)** δ 138.0 (Cq, Ph), 137.4 (Cq, Ph), 128.6 (Ph), 128.5 (Ph), 128.0 (Ph), 127.8 (Ph), 127.8 (Ph), 113.3 (Cq, ketal), 105.5 (CH, C^1), 87.7 (d, $J^{2}_{C\text{-}F}$= 17.0 Hz, Cq, C^4), 85.7 (CH, C^2), 84.5 (CH, C^3), 83.4 (d, $J^{1}_{C\text{-}F}$= 171.8 Hz, CH_2, C^5), 73.6 (CH_2, Bn), 72.5 (CH_2, Bn), 69.6 (d, $J^{3}_{C\text{-}F}$= 8.2 Hz, CH_2, C^6), 27.3 (CH_3, ketal), 26.9 (CH_3, ketal). **^{19}F NMR (471 MHz, Chloroform-*d*)** δ -233.5, (t, $J^{2}_{F\text{-}H}$ = 47.2 Hz). **IR (neat) σ** 2939, 1454, 1374, 1209, 1164, 1069, 1009, 735, 696. **HRMS (ESI$^+$) Calcd for $C_{23}H_{27}O_5NaF$ [M+Na]$^+$**= 425.1740; found= 425.1740. $[\alpha]^{25}_{D}$= -34.4 (c 1.0, $CHCl_3$).

3,6-di-*O*-benzyl-4-*C*-fluoromethyl-β-L-arabinofuranose, **12**

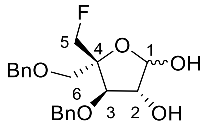

A solution of **11** (203 mg, 0.5 mmol, 1 equiv) in a mixture of acetic acid, water and tetrahydrofuran (8/1/1, 10 mL) was heated at 80 °C for 6 h. The mixture was cooled to room temperature and then concentrated under reduced pressure. Ethyl acetate (10 mL) was added and the organic layer was washed with $NaHCO_3$ (10 mL). The organic layer was dried over $MgSO_4$, filtered and concentrated. The crude **12** (130 mg) was used as such in the next step.

^{1}H NMR (500 MHz, Chloroform-*d*) δ 7.40 – 7.27 (m, 10H, Ph), 5.27 (d, $J^{3}_{H\text{-}H}$ = 7.3 Hz, 1H, H^1α, H^1β), 4.79 – 4.39 (m, 5H, Bnβ, Bnα, H^5α, H^5β), 4.13 – 4.08 (m, 1.65H, H^2β, H^3α, H^3β), 4.06 (s, 0.35H, H^2α), 3.69 (dd, $J^{2}_{H\text{-}H}$ = 10.1 Hz, $J^{4}_{H\text{-}F}$ = 2.0 Hz, 0.35H, H^6aα), 3.66 (dd, $J^{2}_{H\text{-}H}$ = 10.1 Hz, $J^{4}_{H\text{-}F}$ 2.3 Hz, 0.35H, H^6bα), 3.62 (dd, $J^{2}_{H\text{-}H}$ = 9.8 Hz, $J^{4}_{H\text{-}F}$ 1.7 Hz, 0.65H, H^6aβ), 3.53 (dd, $J^{2}_{H\text{-}H}$ = 9.8 Hz, $J^{4}_{H\text{-}F}$ = 1.8 Hz, 0.65H, H^6bβ). **^{13}C NMR (126 MHz, Chloroform-*d*)** δ 137.8 (Cq, Phβ), 137.0 (Cq, Phα), 136.6 (Cq, Phβ), 136.4 (Cq, Phα), 128.9 (Ph), 128.8 (Ph), 128.6 (Ph), 128.5 (Ph), 128.4 (Ph), 128.2 (Ph), 128.1 (Ph), 128.0 (Ph),

127.8 (Ph), 104.5 (CH, $C^1\alpha$), 96.4 (CH, $C^1\beta$), 87.1 (d, $J^{2}_{C\text{-}F}$ = 17.4 Hz, Cq, $C^4\alpha$), 85.5 (CH, $C^2\alpha$), 84.1 (d, $J^{1}_{C\text{-}F}$ = 173.6 Hz, CH_2, $C^5\alpha$), 83.8 (CH, $C^2\beta$), 83.6 (d, $J^{2}_{C\text{-}F}$ = 16.6 Hz, Cq, $C^4\beta$), 83.2 (d, $J^{1}_{C\text{-}F}$ = 174.2 Hz, CH_2, $C^5\beta$), 78.6 (CH, $C^3\alpha$), 76.9 (CH, $C^3\beta$), 74.3 (CH_2, Bnα), 74.2 (CH_2, Bnβ), 73.4 (CH_2, Bnα), 72.8 (CH_2, Bnβ), 71.9 (d, $J^{3}_{C\text{-}F}$ = 6.0 Hz, CH_2, $C^6\alpha$), 71.4 (d, $J^{3}_{C\text{-}F}$ = 6.8 Hz, CH_2, $C^6\beta$). **^{19}F NMR (471 MHz, Chloroform-*d*)** δ -228.1 (d, $J^{2}_{F\text{-}H}$ = 46.9 Hz), -232.7 (d, $J^{2}_{F\text{-}H}$ = 46.9 Hz).

1,3-di-*O*-benzyl-2-*C*-fluoromethyl-D-erythritol, **13**

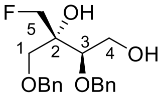

To a solution of **12** (102 mg, 0.28 mmol, 1 equiv) in water (5 mL) and methanol (5 mL) was added $NaIO_4$ (120 mg, 0.56 mmol, 2 equiv) at 0 °C. The reaction mixture was stirred at room temperature for 3 h. Then $NaBH_4$ (21 mg, 0.56 mmol, 2 equiv) was added and the mixture stirred at room temperature for 1 h. The mixture was concentrated under reduced pressure. NH_4Cl sat. (10 mL) was added and the aqueous layer was extracted with diethyl ether (3x10 mL). The combined organic layers were dried over $MgSO_4$, filtrated and concentrated. The crude residue was purified by silica gel column chromatography (petroleum ether/diethyl ether, 8/2) to afford **13** as a colorless oil (82.5 mg, 0.25 mmol, 88%).

^{1}H NMR (500 MHz, Chloroform-*d*) δ 7.39 – 7.28 (m, 10H, Ph), 4.69 (d, $J^{2}_{H\text{-}H}$= 11.5 Hz, 1H, Bn), 4.61 – 4.52 (m, 4H, H^5a, 3xH^{Bn}), 4.48 (dd, $J^{2}_{H\text{-}F}$= 36.8 Hz, $J^{2}_{H\text{-}H}$= 9.7 Hz, 1H, H^5b), 3.90 (dd, $J^{2}_{H\text{-}H}$= 12.2 Hz, $J^{3}_{H\text{-}H}$= 4.7 Hz, 1H, H^4a), 3.79 (dd, $J^{2}_{H\text{-}H}$= 12.2 Hz, $J^{3}_{H\text{-}H}$= 4.2 Hz, 1H, H^4b), 3.69 – 3.62 (m, 2H, H^3, H^1a), 3.60 (dd, $J^{2}_{H\text{-}H}$= 9.6 Hz, $J^{4}_{H\text{-}F}$= 2.1 Hz, 1H, H^1b). **^{13}C NMR (126 MHz, Chloroform-*d*)** δ 137.9 (Cq, Ph), 137.5 (Cq, Ph), 128.7 (Ph), 128.7 (Ph), 128.2 (Ph), 128.2 (Ph), 128.1 (Ph), 128.0 (Ph), 83.8 (d, $J^{1}_{C\text{-}F}$= 173.7 Hz, CH_2, C^5), 79.0 (d, $J^{3}_{C\text{-}F}$= 3.2 Hz, CH, C^3), 76.0 (d, $J^{2}_{C\text{-}F}$= 16.7 Hz, Cq, C^2), 73.9 (CH_2, Bn), 73.4 (CH_2, Bn), 69.9 (d, $J^{3}_{C\text{-}F}$= 5.6 Hz, CH_2, C^1), 60.6 (CH_2, C^4). **^{19}F NMR (471 MHz, Chloroform-*d*)** δ -233.9 (t, $J^{2}_{F\text{-}H}$= 47.5 Hz). **IR (neat)** σ 3408, 2873, 1454, 1092, 1016, 734, 696. **HRMS (ESI$^+$) Calcd for $C_{19}H_{23}O_4FSi$ [M+Na]$^+$**= 357.1478; found= 357.1479. $[\alpha]^{25}_{D}$= -21.2° (c 1.0, $CHCl_3$).

1,3-di-*O*-benzyl-2,4-*O*-bis(dicyanoethylphosphate)-2-*C*-fluoromethyl-D-erythritol, **14**

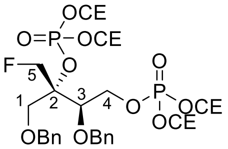

Under argon atmosphere, to a solution of **13** (138 mg, 0.41 mmol, 1 equiv) in dichloromethane (4 mL) was added 5-phenyl-1*H*-tetrazole (363 mg, 2.48 mmol, 6 equiv) and dicyanoethyl diisopropylphosphoramidite (320 µL, 1.22 mmol, 3 equiv). The mixture was stirred at room temperature for 24 h. Then, it was cooled to 0 °C and hydrogen peroxide 30% (320 µL, 4.09 mmol, 10 equiv) was added. The mixture was warmed to room temperature and stirred for 2 h. Then, dichloromethane (10 mL) was added and the organic layer was washed with $Na_2S_2O_4$ 10% (10 mL) and $NaHCO_3$ sat. (10 mL). The aqueous layers were extracted with dichloromethane (2x15 mL). The combined organic layers were dried over $MgSO_4$, filtrated and concentrated. The crude was purified

by a first flash column chromatography (ethyl acetate/methanol, 99/1) and a second (dichloromethane/methanol, 99/1 to 97/3) to afford **14** as a colorless oil (241 mg, 0.34 mmol, 82%).

^{1}H NMR (500 MHz, Chloroform-*d*) δ 7.41 – 7.28 (m, 10H, Ph), 4.83 (dd, $J^{2}_{\text{H-F}}$= 39.2 Hz, $J^{2}_{\text{H-H}}$= 10.5 Hz, 1H, H^{5}a), 4.83 (d, $J^{2}_{\text{H-H}}$= 11.1 Hz, 1H, Bn), 4.75 (dd, $J^{2}_{\text{H-F}}$= 40.1 Hz, $J^{2}_{\text{H-H}}$= 10.5 Hz, 1H, H^{5}b), 4.62 (d, $J^{2}_{\text{H-H}}$= 11.2 Hz, 1H, Bn), 4.58 (d, $J^{2}_{\text{H-H}}$= 11.4 Hz, 1H, Bn), 4.56 – 4.53 (m, 1H, H^{4}a), 4.51 (d, $J^{2}_{\text{H-H}}$= 11.4 Hz, 1H, Bn), 4.41 – 4.33 (m, 1H, H^{4}b), 4.28 – 4.11 (m, 9H, H^{3}, 4xCH_2OP), 3.98 (d, $J^{2}_{\text{H-H}}$= 10.4 Hz, 1H, H^{1}a), 3.80 (d, $J^{2}_{\text{H-H}}$= 10.4 Hz, 1H, H^{1}b), 2.74 – 2.53 (m, 8H, 4xCH_2CN). **^{13}C NMR (126 MHz, Chloroform-*d*) δ** 137.4 (Cq, Ph), 137.2 (Cq, Ph), 128.7 (Ph), 128.7 (Ph), 128.3 (Ph), 128.2 (Ph), 128.2 (Ph), 128.0 (Ph), 116.8 (2xCN), 116.6 (2xCN), 86.7 (dd, $J^{2}_{\text{C-F}}$= 16.7 Hz, $J^{2}_{\text{C-P}}$ 7.8 Hz, Cq, C^{2}), 81.9 (d, $J^{1}_{\text{C-F}}$= 175.6 Hz, CH_2F, C^{5}), 77.6 – 77.5 (m, CH, C^{3}), 74.6 (CH_2, Bn), 73.7 (CH_2, Bn), 68.1 (t, $J^{2}_{\text{C-P}}$= 5.7 Hz, CH_2, C^{4}), 67.7-67.4 (m, CH_2, C^{1}), 62.6 – 62.4 (m, 4xCH_2OP), 20.0 – 19.3 (m, 4xCH_2CN). **^{19}F NMR (471 MHz, Chloroform-*d*) δ** -232.8 (t, $J^{2}_{\text{F-H}}$= 46.9 Hz). **^{31}P NMR (203 MHz, Chloroform-*d*) δ** -2.4 (p, $J^{3}_{\text{P-H}}$= 7.5 Hz), -7.6 (p, $J^{3}_{\text{P-H}}$= 7.8 Hz). **IR (neat)** σ 2970, 1279, 1027, 1001. **HRMS (ESI^{+}) Calcd for $C_{31}H_{38}N_{4}O_{4}P_{2}F$ [M+H]$^{+}$**= 707.2047; found= 707.2043. $[\alpha]^{25}_{D}$= -9.4° (c 1.0, $CHCl_3$).

1,3-di-*O*-benzyl-2,4-*O*-diphosphate-2-*C*-fluoromethyl-D-erythritol, Ammonium salt, **15**

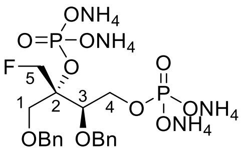

To a solution of **14** (187 mg, 0.26 mmol, 1 equiv) in methanol (1 mL) was added 3 mL of a concentrated solution (35%) of ammonium hydroxide. The mixture was heated to 55 °C and stirred for 24 h. Then, it was concentrated, passed through an ion exchange resin (DOWEX 50WX8 NH_4^{+}) and lyophilised to afford **15** as a white foam.

^{1}H NMR (500 MHz, Deuterium Oxide) δ 7.44 – 7.30 (m, 10H, Ph), 4.86 (d, $J^{2}_{\text{H-H}}$= 11.0 Hz, 1H, Bn), 4.82 (dd, $J^{2}_{\text{H-F}}$= 47.2 Hz, $J^{2}_{\text{H-H}}$= 10.7 Hz, 1H, H^{5}a), 4.71 (dd, $J^{2}_{\text{H-F}}$= 47.5 Hz, $J^{2}_{\text{H-H}}$=10.5 Hz, 1H, H^{5}b), 4.65 (d, $J^{2}_{\text{H-H}}$= 11.7 Hz, 1H, Bn), 4.58 (d, $J^{2}_{\text{H-H}}$= 10.9 Hz, 1H, Bn), 4.53 (d, $J^{2}_{\text{H-H}}$= 11.7 Hz, 1H, Bn), 4.27 (ddd, $J^{2}_{\text{H-H}}$= 11.3 Hz, $J^{3}_{\text{H-P}}$= 5.2 Hz, $J^{3}_{\text{H-H}}$= 2.6 Hz, 1H, H^{4}a), 4.18 – 4.13 (m, 1H, H^{3}), 4.09 – 4.02 (m, 1H, H^{4}b), 3.93 (q, $J^{2}_{\text{H-H}}$= 10.8 Hz, 2H, H^{1}). **^{13}C NMR (126 MHz, Deuterium Oxide) δ** 137.4 (Cq, Ph), 137.3 (Cq, Ph), 128.7 (Ph), 128.7 (Ph), 128.6 (Ph), 128.2 (Ph), 128.2 (Ph), 83.20 (d, $J^{1}_{\text{C-F}}$= 172.2 Hz, CH_2, C^{5}), 81.3 (dd, $J^{2}_{\text{C-F}}$= 16.8 Hz, $J^{2}_{\text{C-P}}$= 7.9 Hz, Cq, C^{2}), 79.5 – 79.2 (m, CH, C^{3}), 74.3 (CH_2, Bn), 73.4 (CH_2, Bn), 68.5 (d, $J^{3}_{\text{C-P}}$= 8.6 Hz, CH_2, C^{1}), 64.7 – 64.5 (m, CH_2, C^{4}). **^{19}F NMR (471 MHz, Deuterium Oxide) δ** -230.3 (t, $J^{2}_{\text{F-H}}$= 47.1 Hz). **^{31}P NMR (203 MHz, Deuterium Oxide) δ** 2.07 (s), -3.50 (s). **IR (neat)** σ 2834, 1454, 1063, 1042, 1014, 975, 946. **HRMS (ESI^{+}) Calcd for $C_{19}H_{26}O_{10}P_{2}F$ [M+H]$^{+}$**= 495.0985; found= 495.0984.

1,3-di-*O*-benzyl-2-*C*-fluoromethyl-D-erythritol 2,4-cyclopyrophosphate, Ammonium salt, **16**

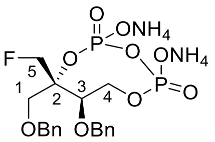

To a solution of **15** (87 mg, 0.15 mmol, 1 equiv) in dimethylformamide (5 mL) was added DCC (189 mg, 0.92 mmol, 6 equiv). The mixture was stirred at room temperature for 5 h and then concentrated under reduced pressure. Water was added to the resulting oil and the heterogenous mixture was filtrated once with wool and a second time with a syringue filter. The crude was purified by reversed phase silica gel (C_{18}) column chromatography (water 100% to methanol 100%) to afford **16** as a white foam (64 mg, 0.12 mmol, 79%). The purified compound was passed through an ion exchange resin (DOWEX 50WX8, NH_4^+) and lyophilized.

^{1}H NMR (500 MHz, Deuterium Oxide) δ 7.50 – 7.33 (m, 10H, Ph), 4.77 (dd, $J^{2}_{H\text{-}F}$= 47 Hz, $J^{2}_{H\text{-}H}$= 10 Hz, 1H, H^5a), 4.72 (d, $J^{2}_{H\text{-}H}$= 11.4 Hz, 1H, Bn), 4.62 (d, $J^{2}_{H\text{-}H}$= 11.9 Hz, 1H, Bn), 4.59 (dd, $J^{2}_{H\text{-}F}$= 47 Hz, $J^{2}_{H\text{-}H}$= 10 Hz, 1H, H^5b) 4.52 (d, $J^{2}_{H\text{-}H}$= 11.9 Hz, 1H, Bn), 4.49 (d, $J^{2}_{H\text{-}H}$= 11.4 Hz, 1H, Bn), 4.35 (td, $J^{2}_{H\text{-}H}$= 13.6 Hz, $J^{3}_{H\text{-}H}$= 6.6 Hz, 1H, H^4a), 4.20 (t, $J^{2}_{H\text{-}H}$= 12.6 Hz, 1H, H^4b), 4.07 – 4.00 (m, 2H, H^1a, H^3), 3.80 (app d, $J^{2}_{H\text{-}H}$= 10.6 Hz, 1H, H^1b). **^{13}C NMR (126 MHz, Deuterium Oxide) δ** 137.1 (Cq, Ph), 136.4 (Cq, Ph), 129.0 (Ph), 128.8 (Ph), 128.7 (Ph), 128.6 (Ph), 128.4 (Ph), 128.3 (Ph), 83.5 (dd, $J^{1}_{C\text{-}F}$= 172.7 Hz, $J^{3}_{C\text{-}P}$= 7.5 Hz, CH_2, C^5), 82.5 (dd, $J^{2}_{C\text{-}F}$= 16.9 Hz, $J^{2}_{C\text{-}P}$= 8.5 Hz, Cq, C^2), 75.9 (CH, C^3), 73.4 (CH_2, Bn), 72.4 (CH_2, Bn), 68.4 (d, $J^{3}_{C\text{-}F}$= 4.9 Hz, CH_2, C^1), 62.8 (CH_2, C^4). **^{19}F NMR (471 MHz, Deuterium Oxide) δ** -233.7 (t, $J^{2}_{F\text{-}H}$= 46.9 Hz). **^{31}P{1H} NMR (203 MHz, Deuterium Oxide) δ** -10.8 (d, $J^{2}_{P\text{-}P}$= 23 Hz), -16.7 (d, $J^{2}_{P\text{-}P}$= 23 Hz). **IR (neat)** σ 3386, 1635, 1454, 1241, 1075. **HRMS (ESI$^+$) Calcd for $C_{19}H_{24}O_9P_2F$ [M+H]$^+$**= 477.0880; found= 477.0878. $[\alpha]^{25}_{D}$= -24.7° (c 1.0, H_2O).

2-*C*-fluoromethyl-D-erythritol 2,4-cyclopyrophosphate, Ammonium salt, **8**

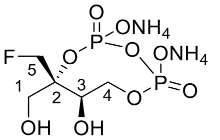

Under hydrogen atmosphere, to a solution of **16** (24 mg, 0.046 mmol, 1 equiv) in methanol (3 mL) was added Pd/C 10% (24 mg) and formic acid (3 drops). The mixture was stirred at room temperature for 18 h. Then, it was filtrated over dicalite and concentrated under reduced pressure. The crude was purified by reversed phase silica gel (C_{18}) column chromatography (water 100%) to afford **8** as a white foam (14 mg, 0.042 mmol, 91%). The purified compound was passed through an ion exchange resin (DOWEX 50WX8, NH_4^+) and lyophilized.

^{1}H NMR (500 MHz, Deuterium Oxide) δ 4.74 (ddd, $J^{2}_{H\text{-}F}$= 46.8 Hz, $J^{2}_{H\text{-}F}$= 36.9 Hz, $J^{2}_{H\text{-}H}$= 9.6 Hz, 1H, H^5), 4.39 (ddd, $J^{3}_{H\text{-}P}$= 12.9 Hz, $J^{2}_{H\text{-}H}$= 11.5 Hz, $J^{3}_{H\text{-}H}$= 1.7 Hz, 1H, H^4a), 4.20 – 4.09 (m, 1H, H^4b), 4.06 – 4.03 (m, 2H, H^1a, H^3), 3.80 (dd, $J^{2}_{H\text{-}H}$= 12.9 Hz, $J^{4}_{H\text{-}P}$= 2.5 Hz, 1H, H^1b). **^{13}C NMR (126 MHz, Deuterium Oxide)** δ 83.9 (dd, $J^{2}_{C\text{-}F}$= 18.4 Hz, $J^{2}_{C\text{-}P}$ = 8.0 Hz, Cq, C^2), 81.1 (dd, $J^{1}_{C\text{-}F}$= 171.4 Hz, $J^{3}_{C\text{-}P}$ = 6.8 Hz, CH_2, C^5), 67.9 (CH, C^3), 65.6 (d, $J^{2}_{C\text{-}P}$= 6.4 Hz, CH_2, C^4), 60.7 – 60.6 (m, CH_2, C^1). **^{19}F NMR (471 MHz, Deuterium Oxide)** δ -233.1 (t, $J^{2}_{F\text{-}H}$= 46.7 Hz). **^{31}P{1H} NMR (203 MHz, Deuterium Oxide)** δ -10.9 (d, $J^{2}_{P\text{-}P}$= 23 Hz), -14.9 (d, $J^{2}_{P\text{-}P}$= 23 Hz). **IR (neat) σ** 3237, 1655, 1444, 1239, 1124, 1051, 936. **HRMS (ESI$^-$) Calcd for $C_5H_{10}O_9P_2F$ [M-H]$^-$**= 294.9784; found= 294.9788. $[\alpha]^{25}_{D}$= -17.7° (c 0.8, H_2O).

NMR spectra of compounds 10-16 and 8

^{1}H-NMR spectrum of 10 ($CDCl_3$, 500 MHz)

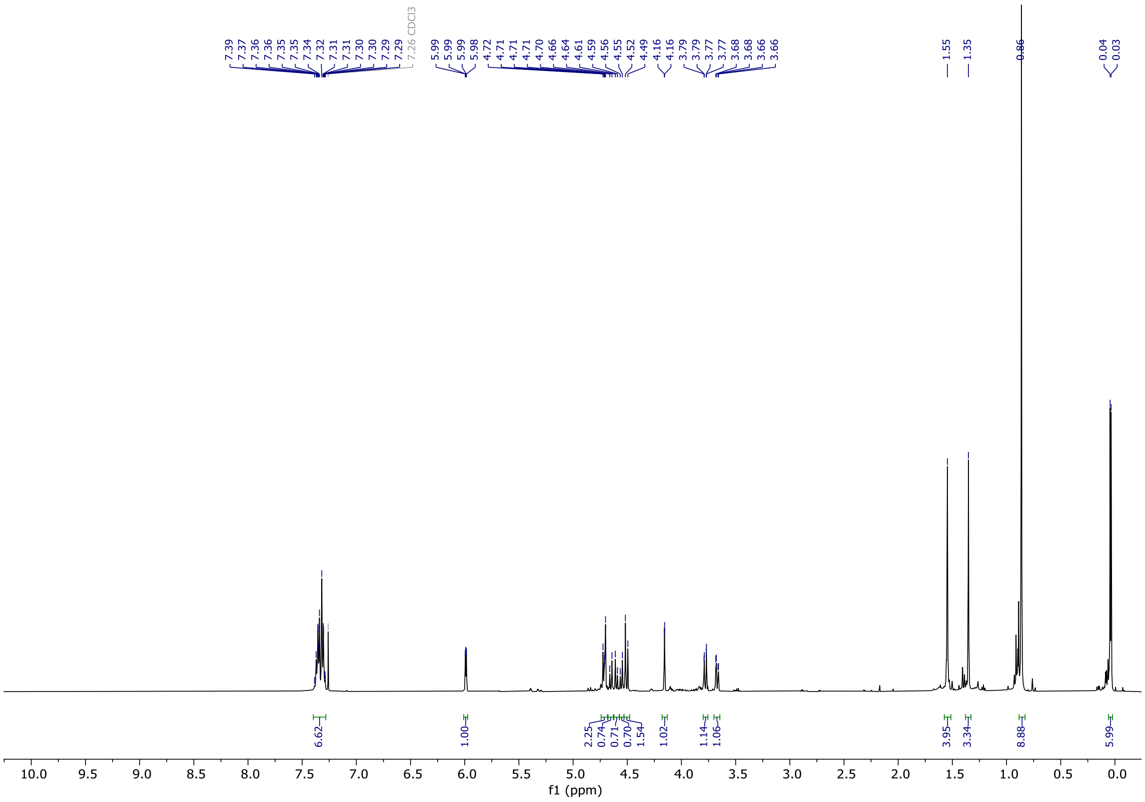

^{13}C-NMR spectrum of 10 ($CDCl_3$, 126 MHz)

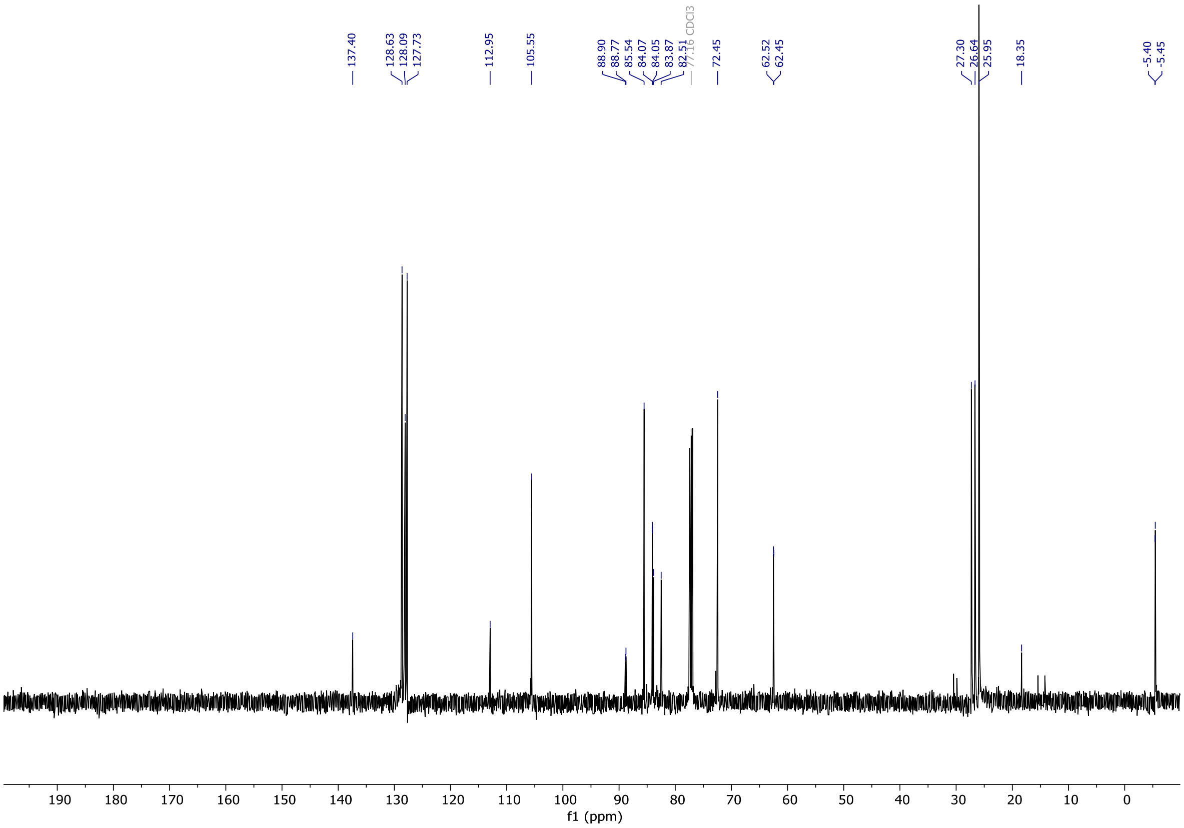

^{19}F-NMR spectrum of 10 ($CDCl_3$, 471 MHz)

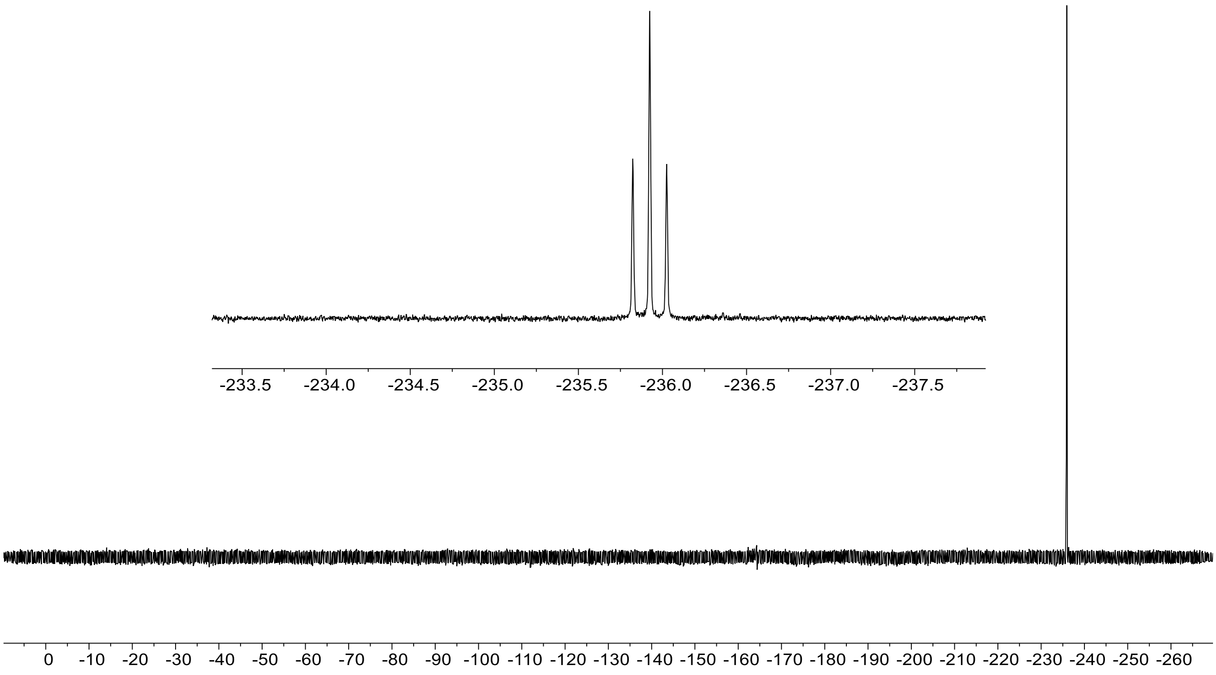

^{1}H-NMR spectrum of 11 (CDCl$_3$, 500 MHz)

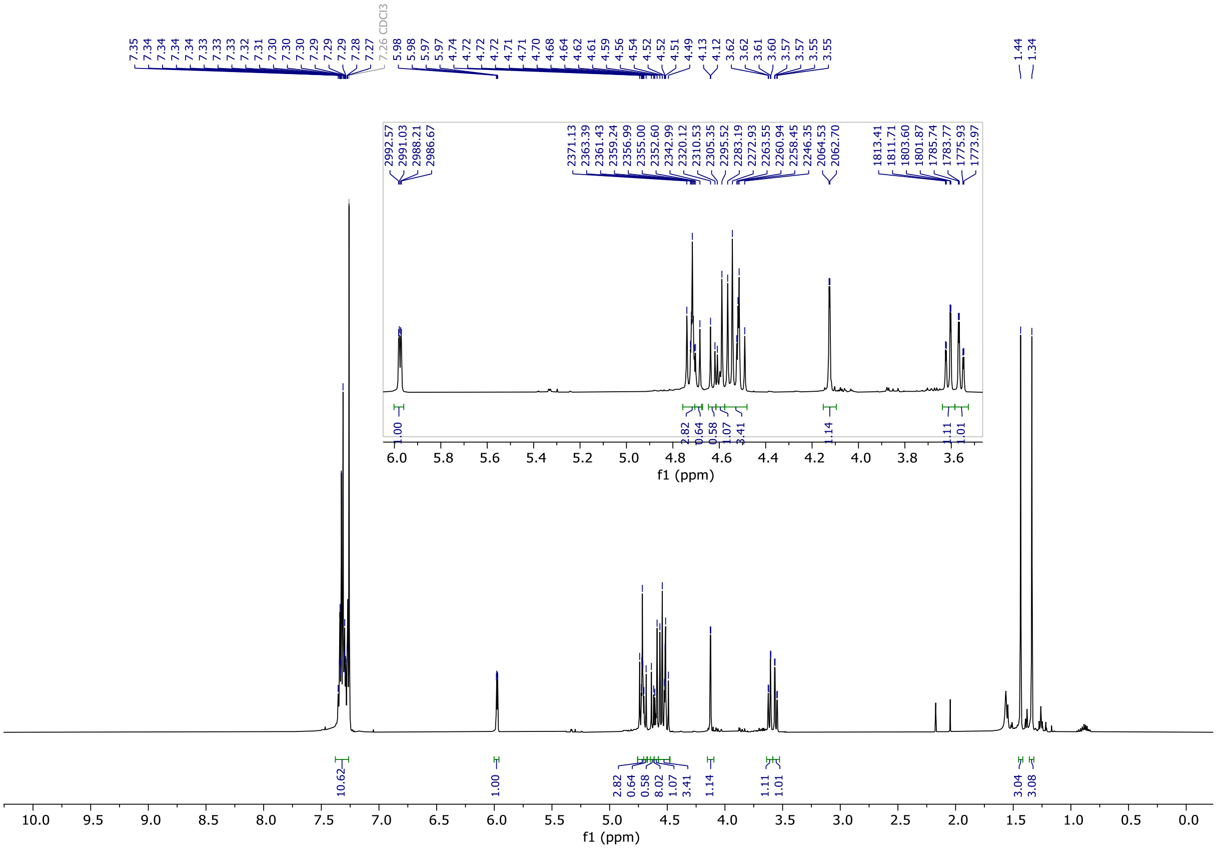

^{13}C-NMR spectrum of 11 (CDCl$_3$, 126 MHz)

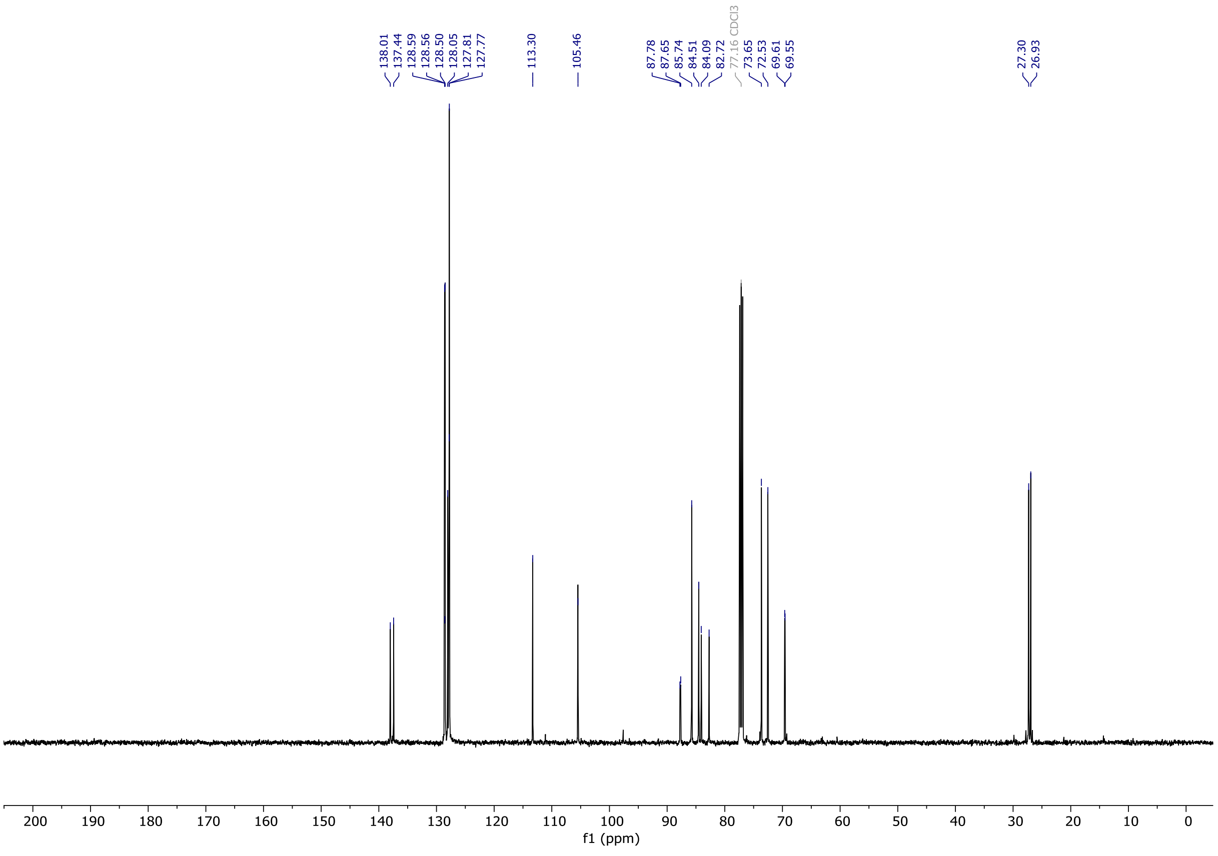

^{19}F-NMR spectrum of 11 ($CDCl_3$, 471 MHz)

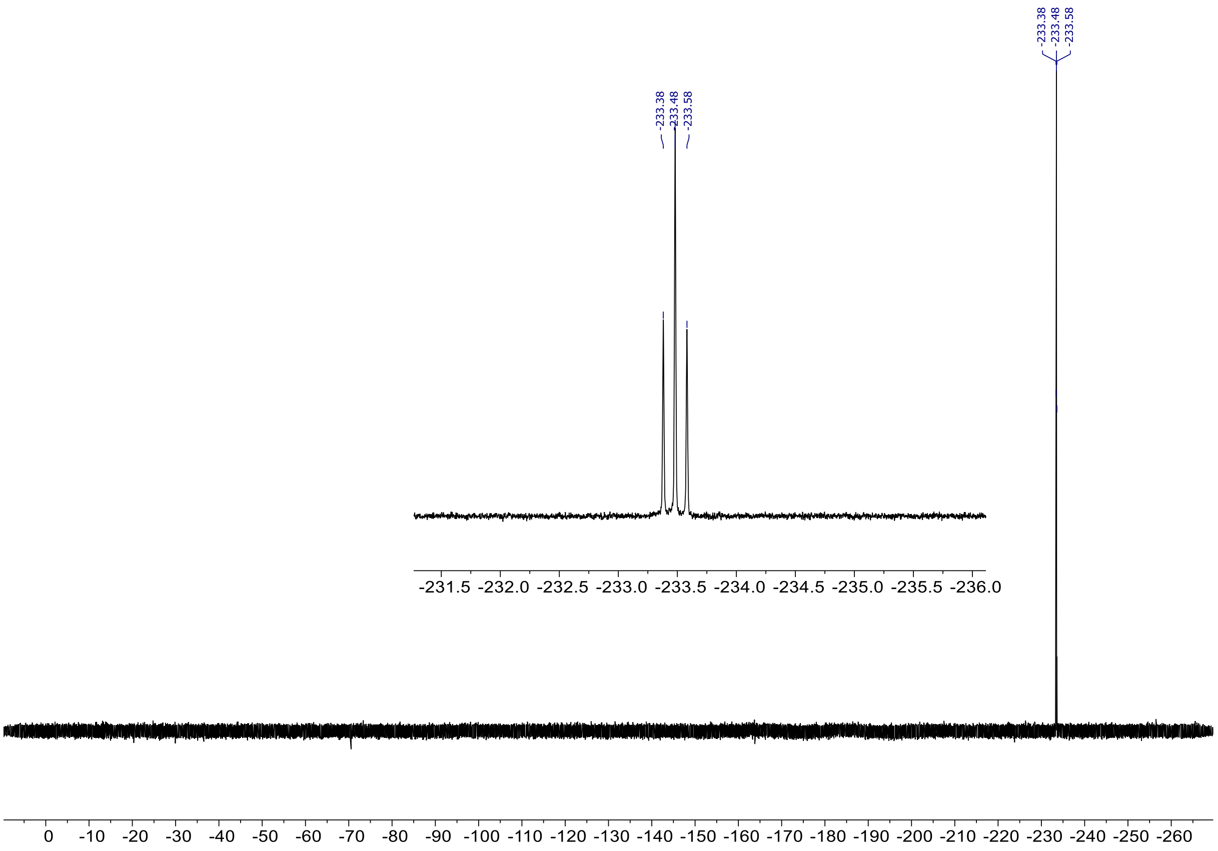

^{1}H-NMR spectrum of 13 ($CDCl_3$, 500 MHz)

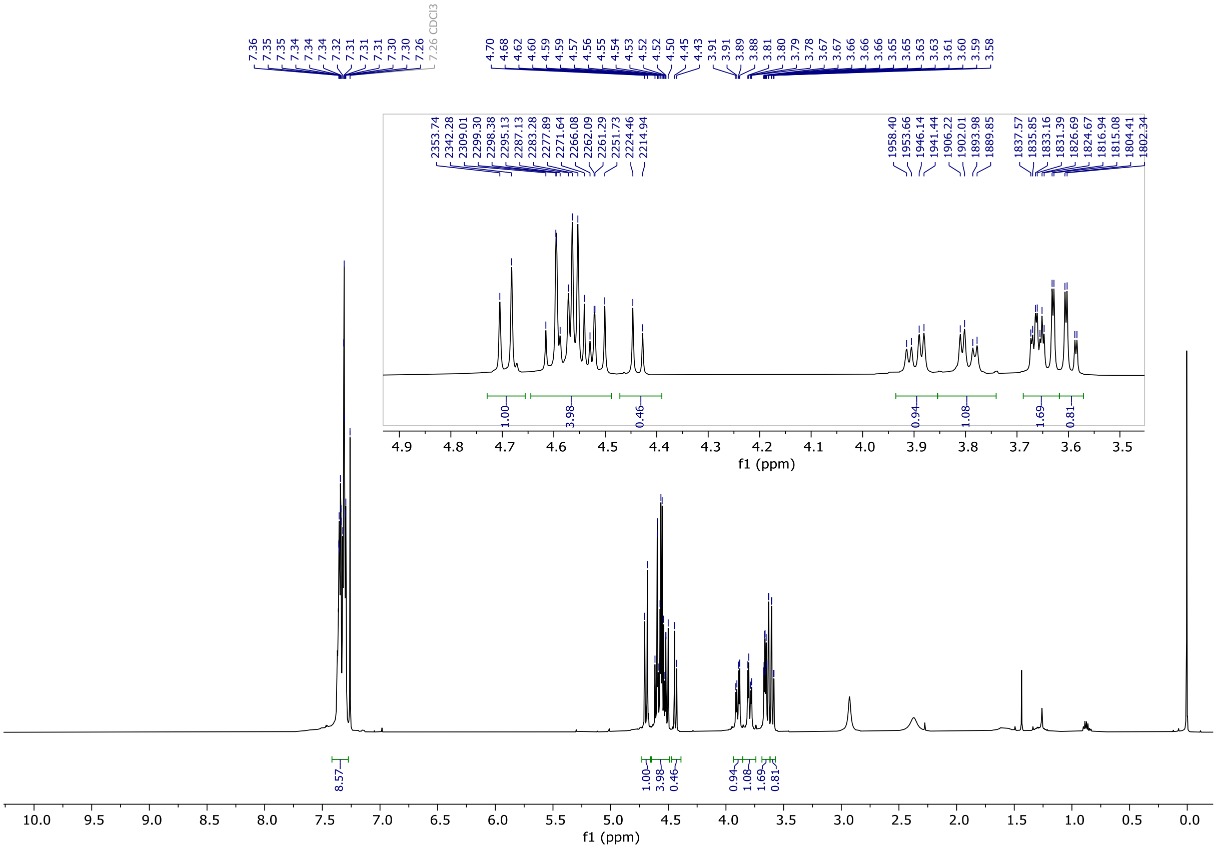

^{13}C-NMR spectrum of 13 ($CDCl_3$, 126 MHz)

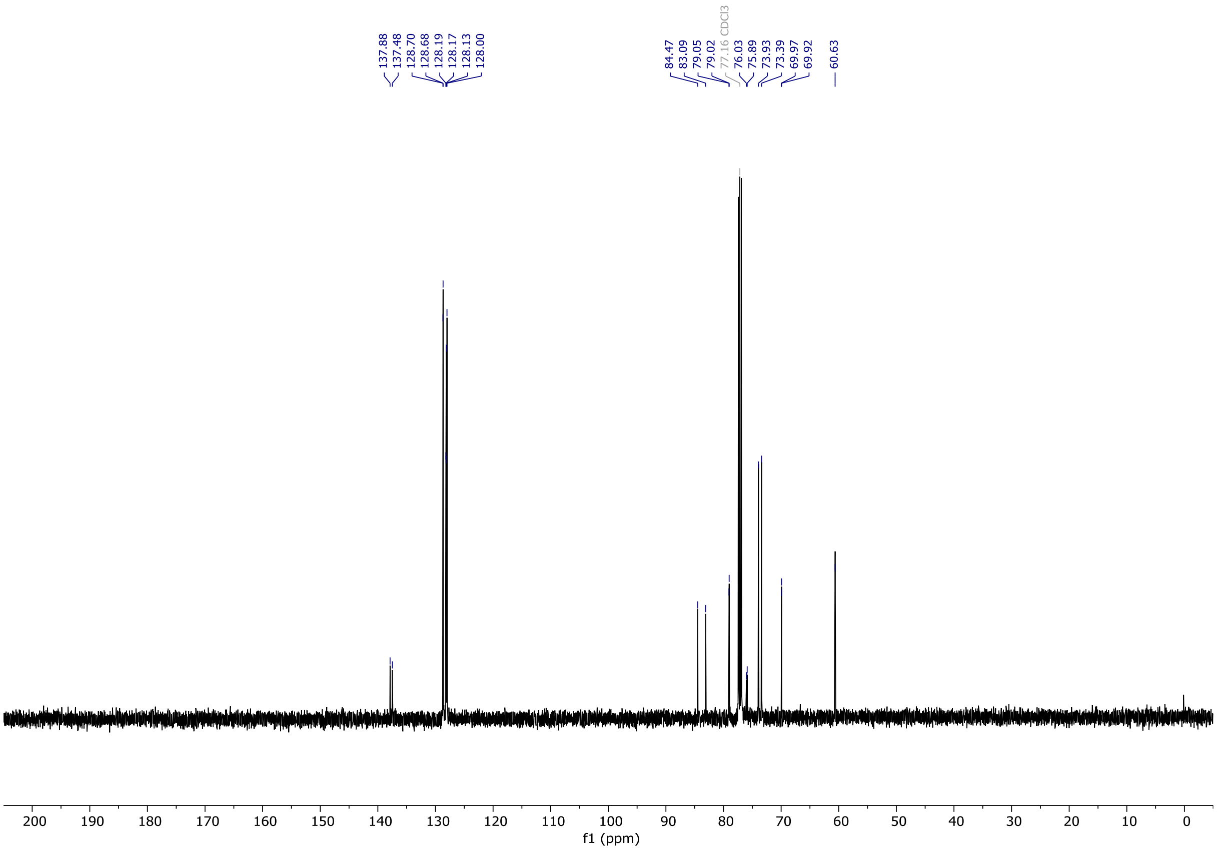

^{19}F-NMR spectrum of 13 ($CDCl_3$, 471 MHz)

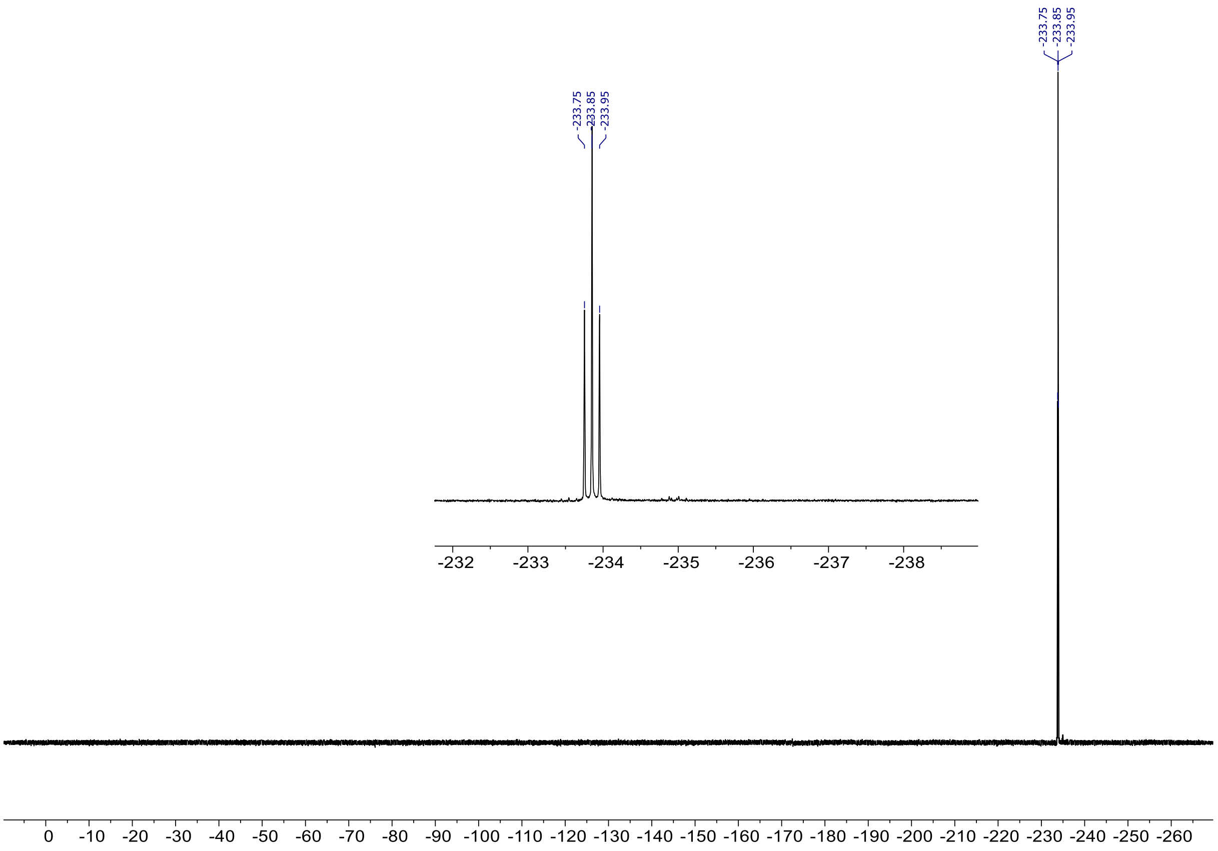

^{1}H-NMR spectrum of 14 (CDCl$_3$, 500 MHz)

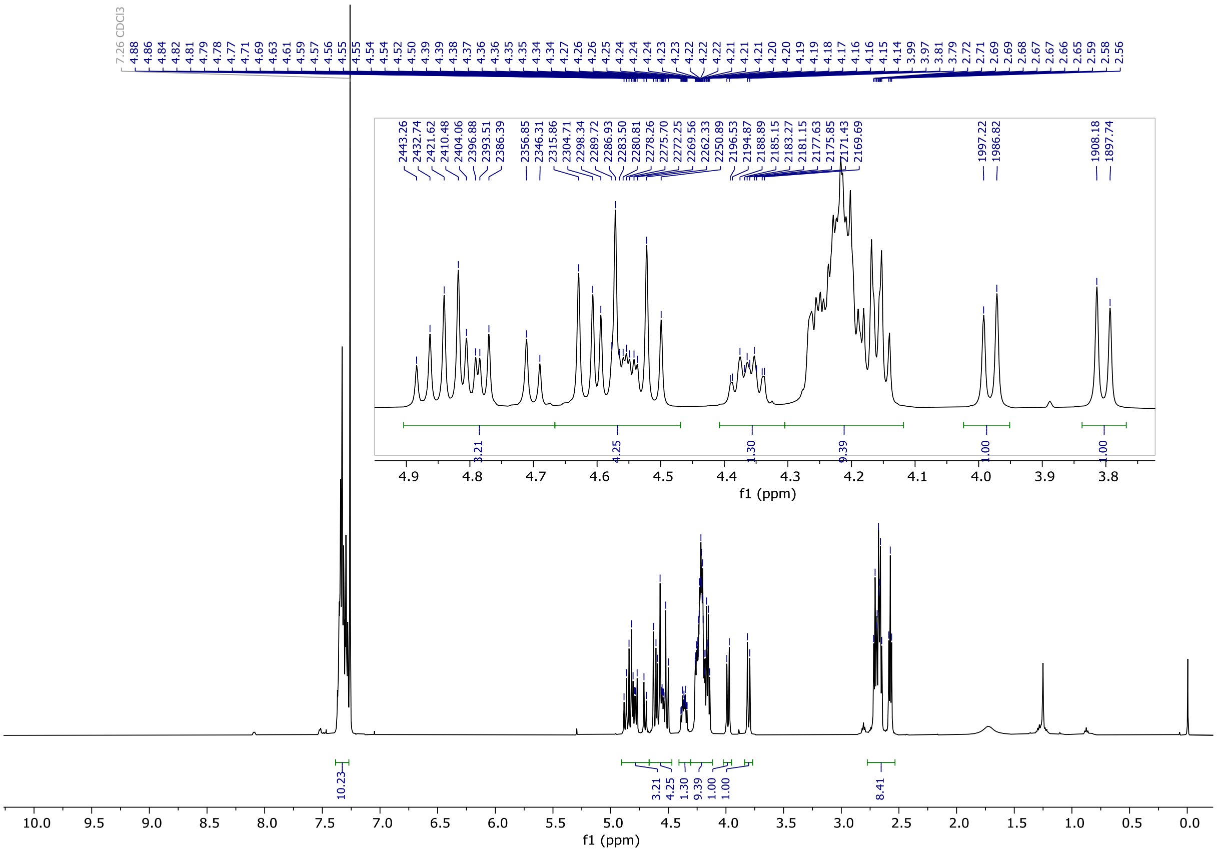

^{13}C-NMR spectrum of 14 (CDCl$_3$, 126 MHz)

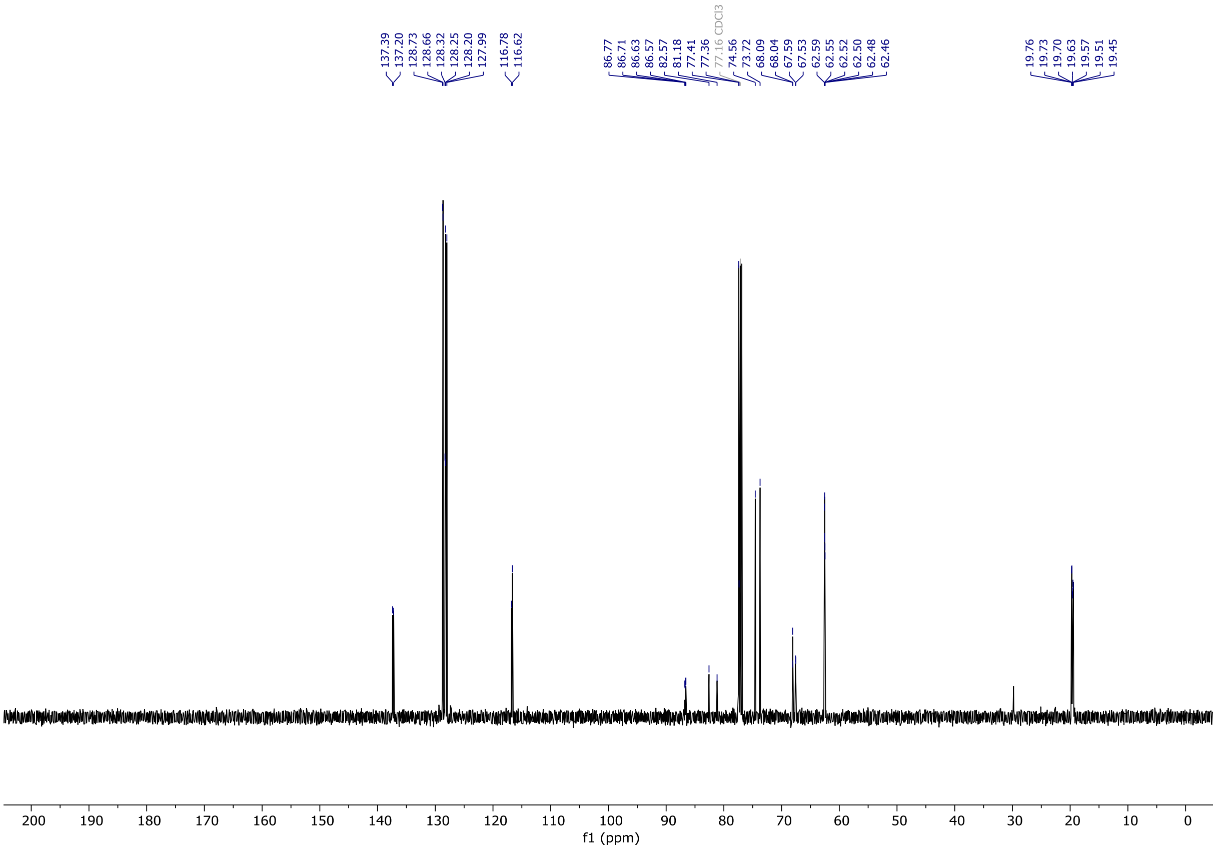

^{19}F-NMR spectrum of 14 ($CDCl_3$, 471 MHz)

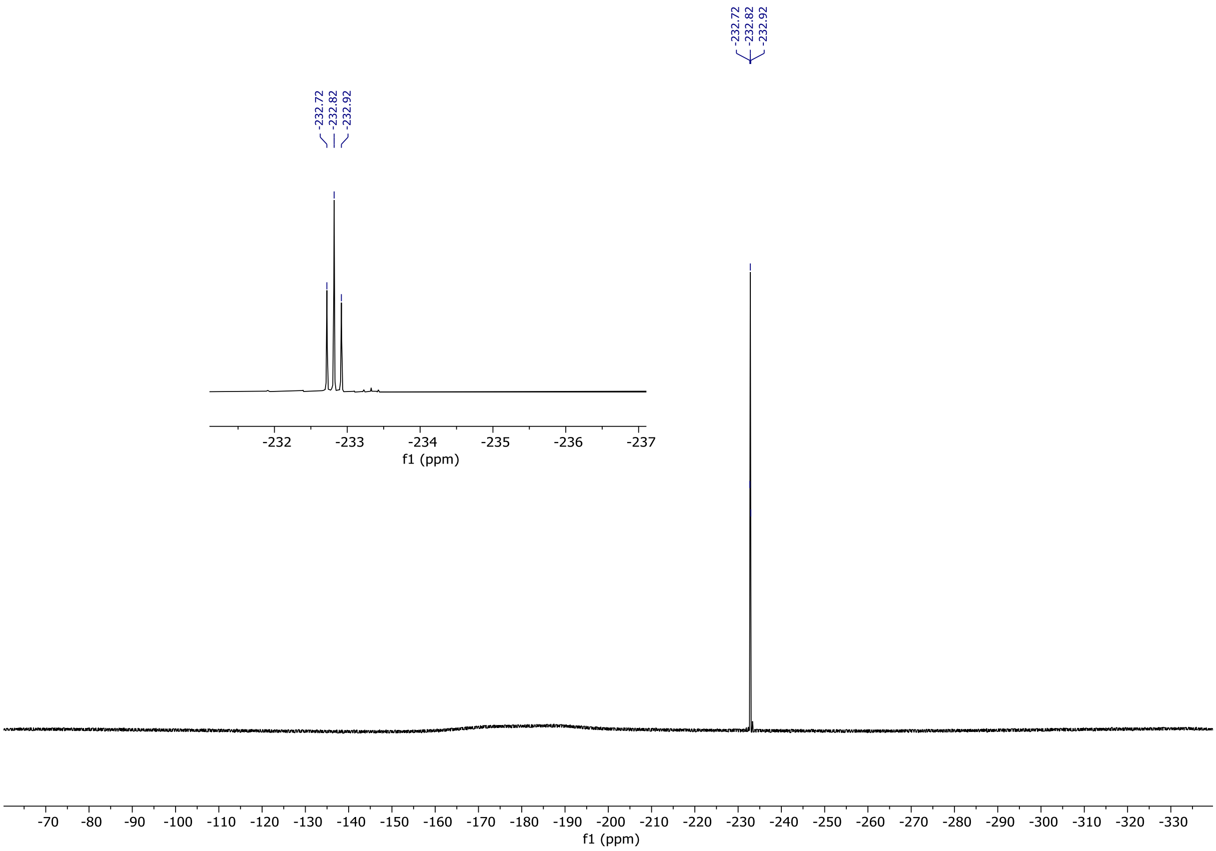

^{31}P-NMR spectrum of 14 ($CDCl_3$, 203 MHz)

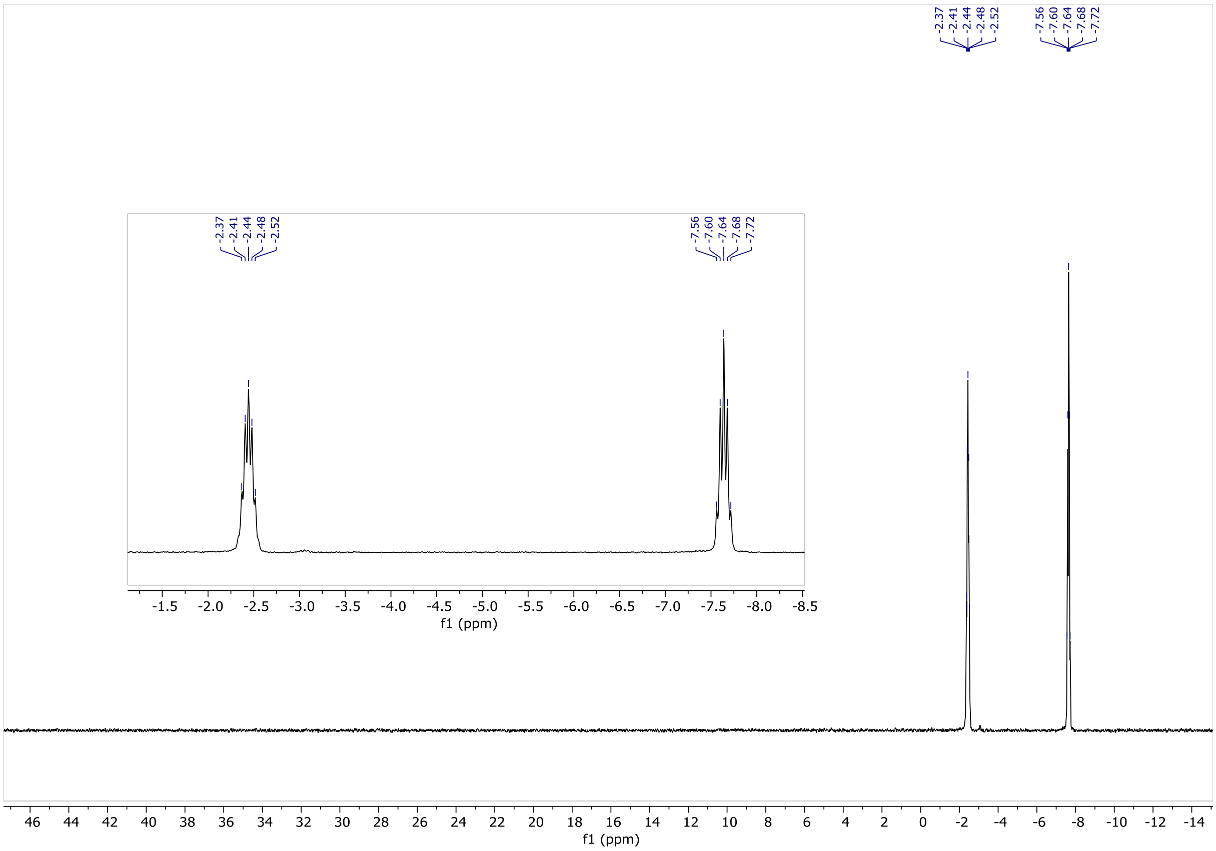

^{1}H-NMR spectrum of 15 (D_2O, 500 MHz)

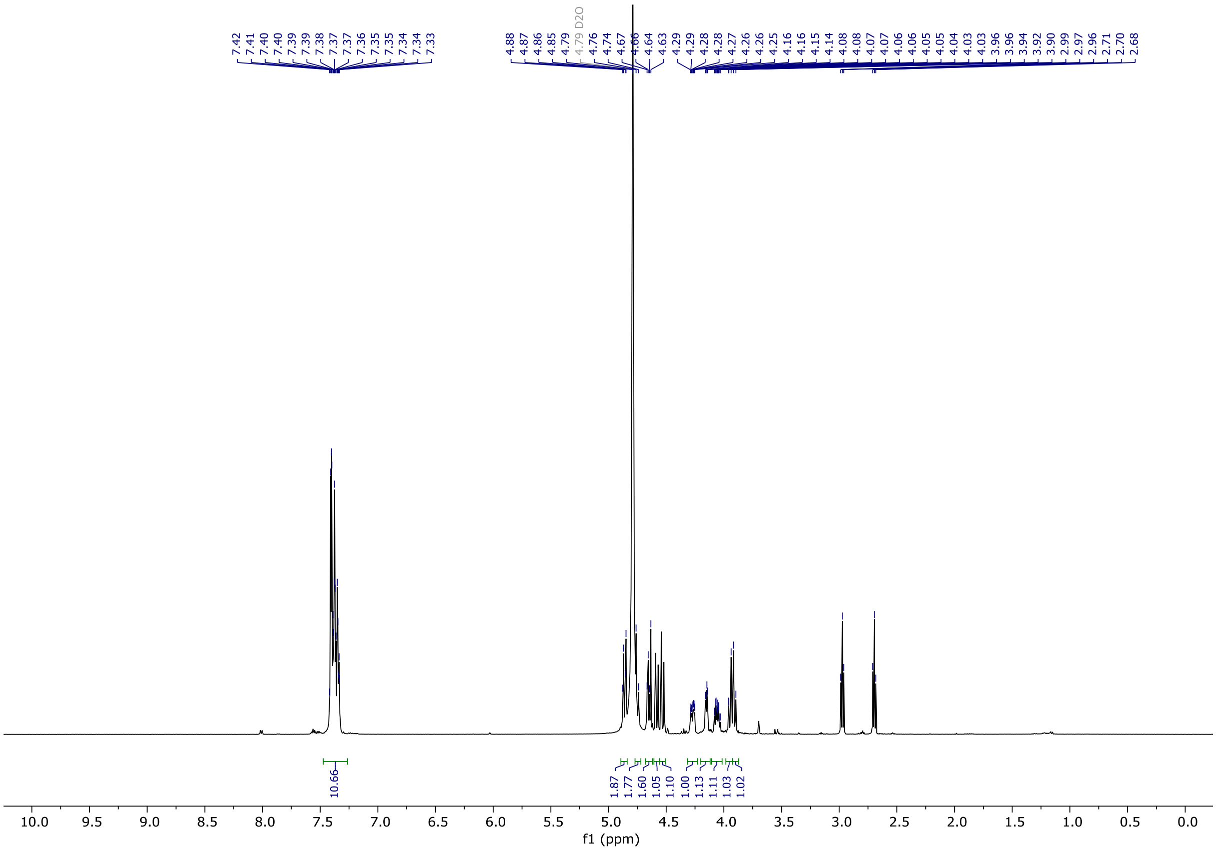

^{13}C-NMR spectrum of 15 (D_2O, 126 MHz)

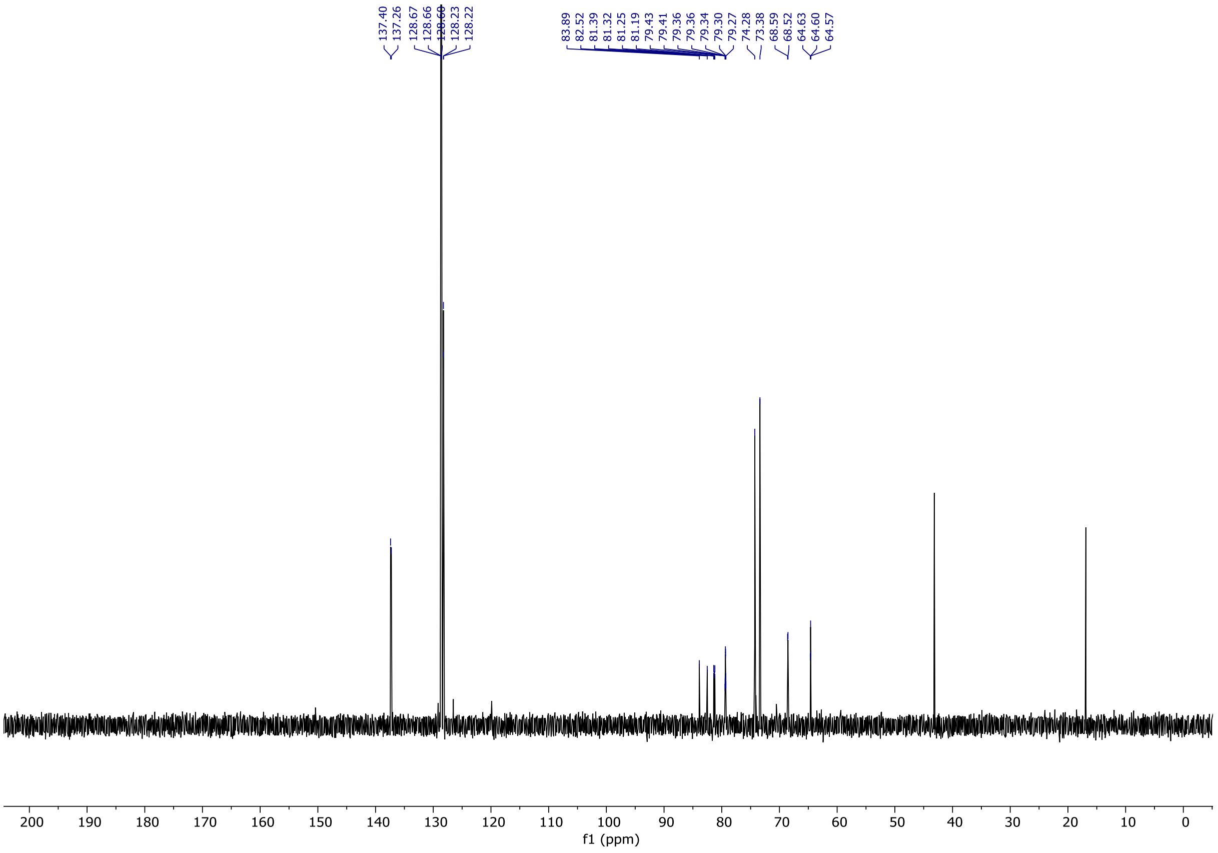

^{19}F-NMR spectrum of 15 (D_2O, 471 MHz)

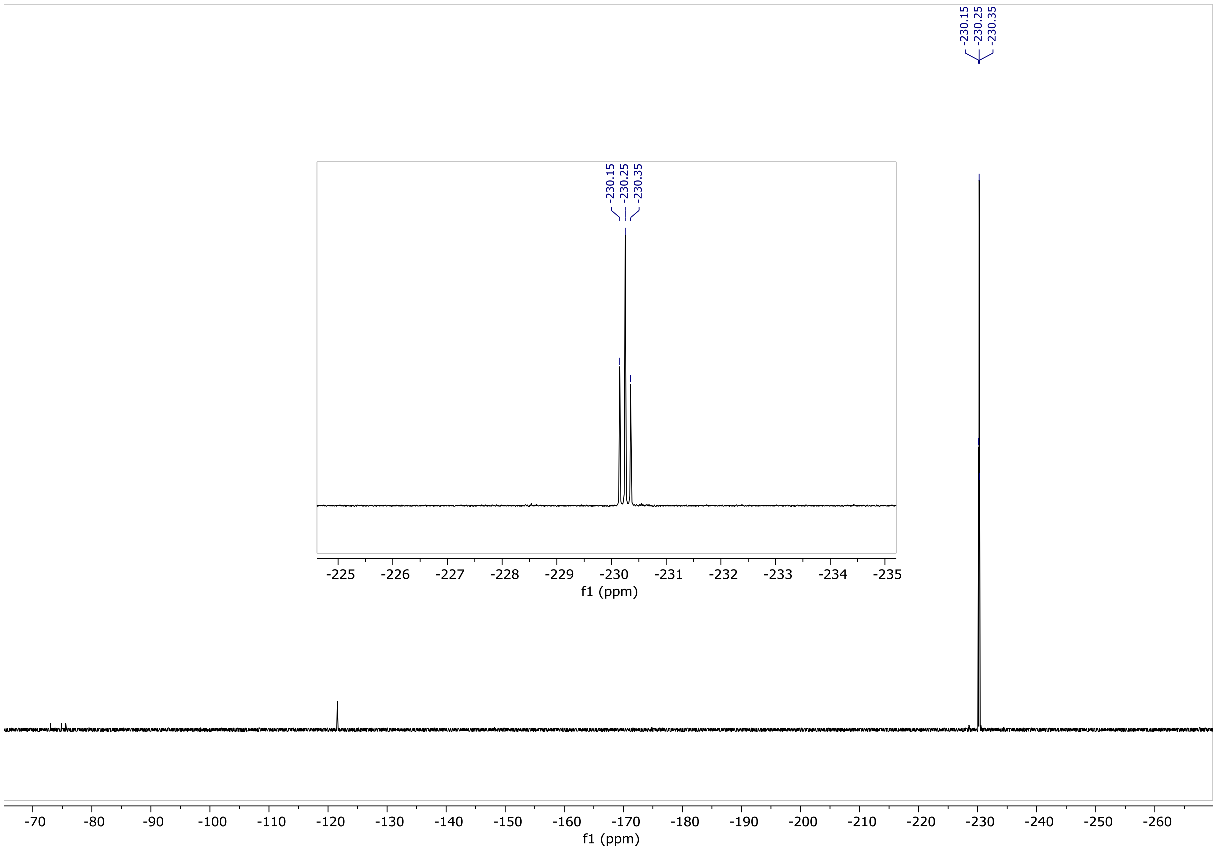

^{31}P-NMR spectrum of 15 (D_2O, 203 MHz)

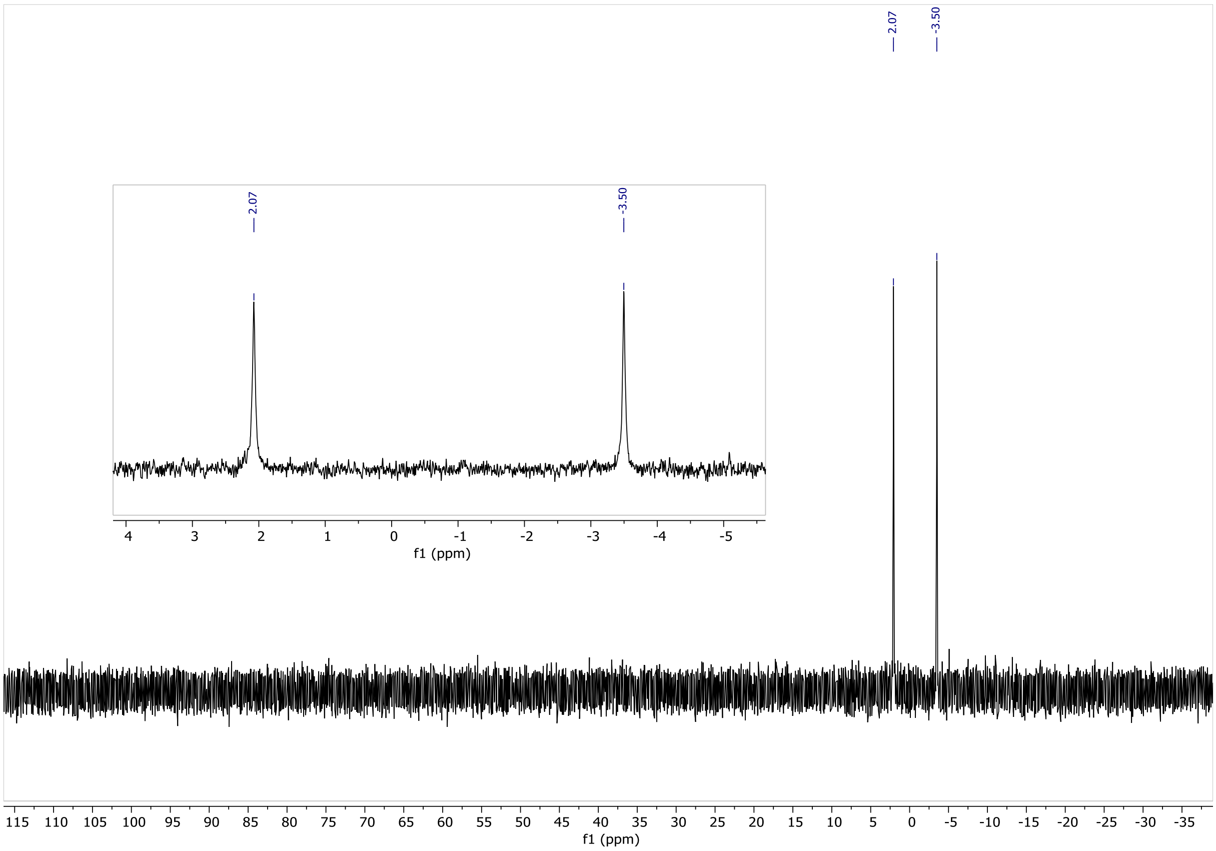

^{1}H-NMR spectrum of 16 (D_2O, 500 MHz)

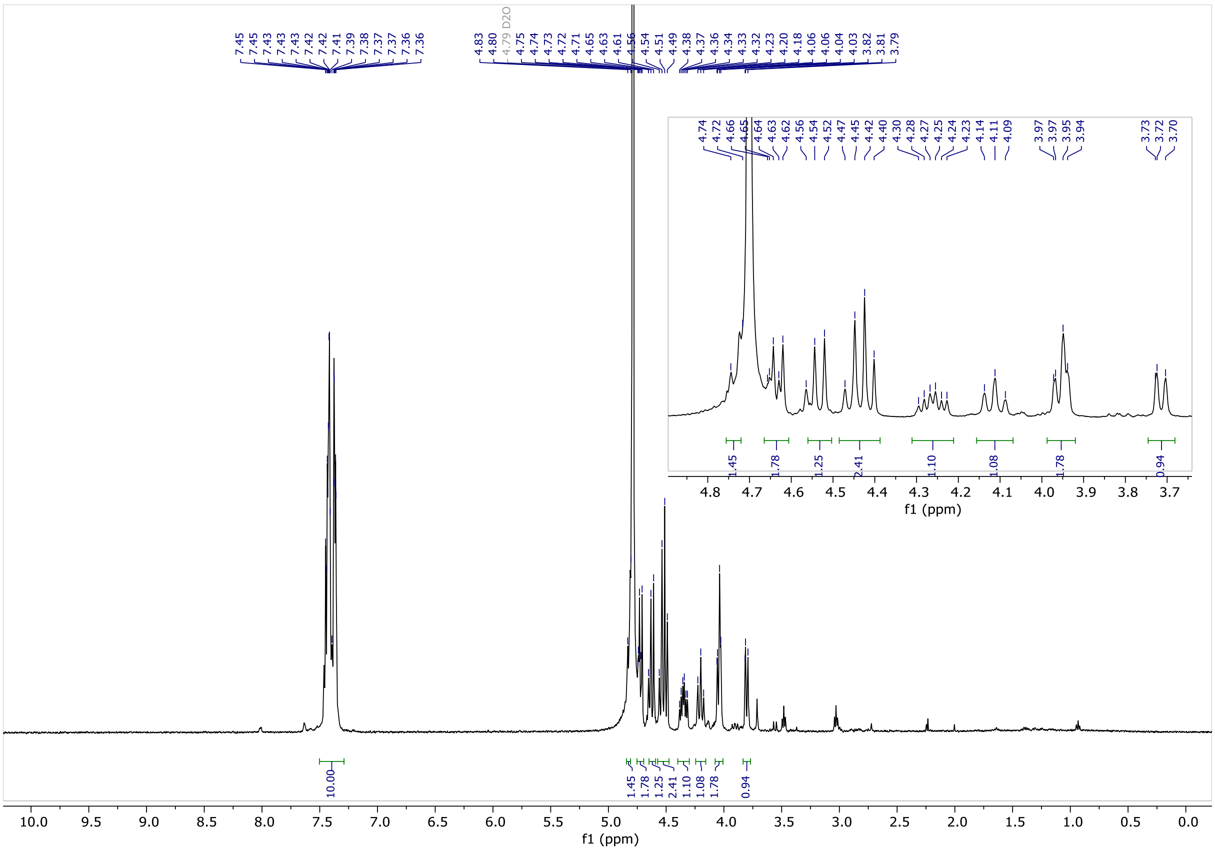

^{13}C-NMR spectrum of 16 (D_2O, 126 MHz)

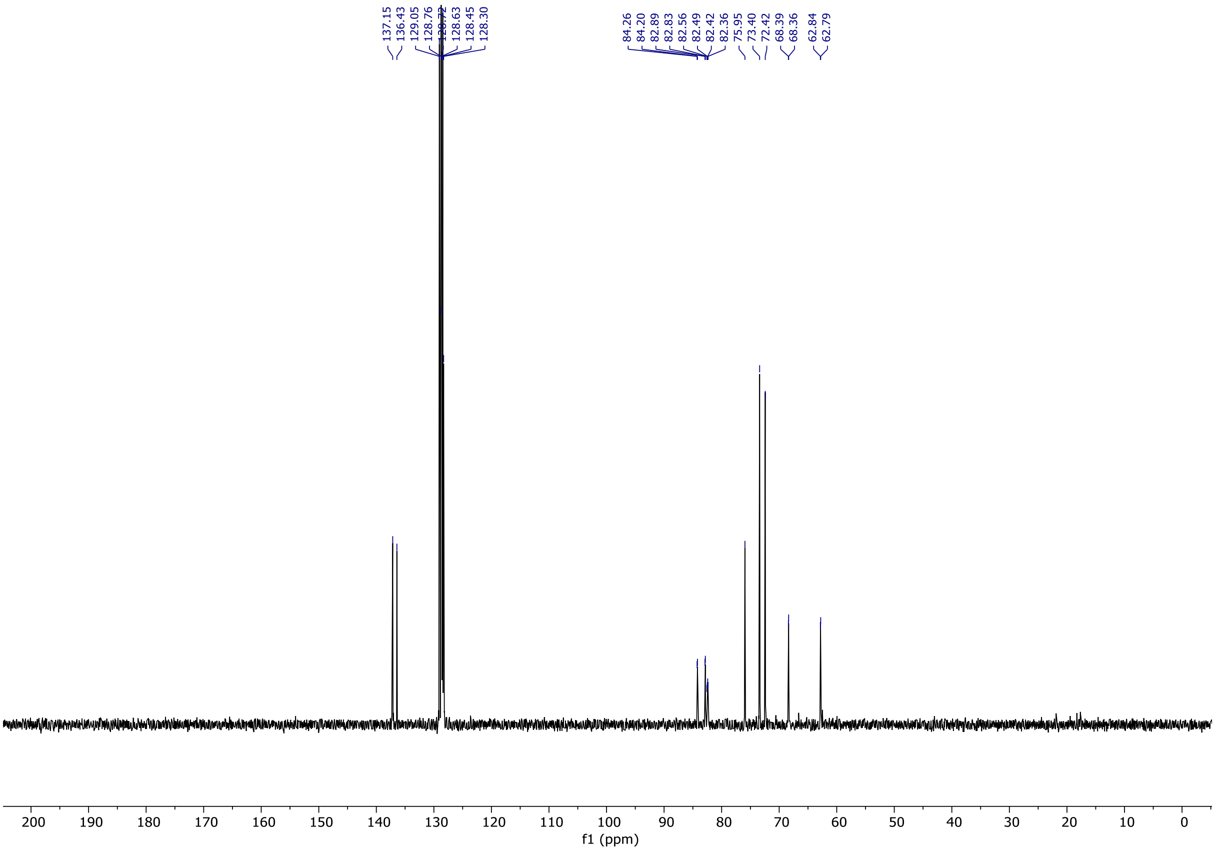

^{19}F-NMR spectrum of 16 (D_2O, 471 MHz)

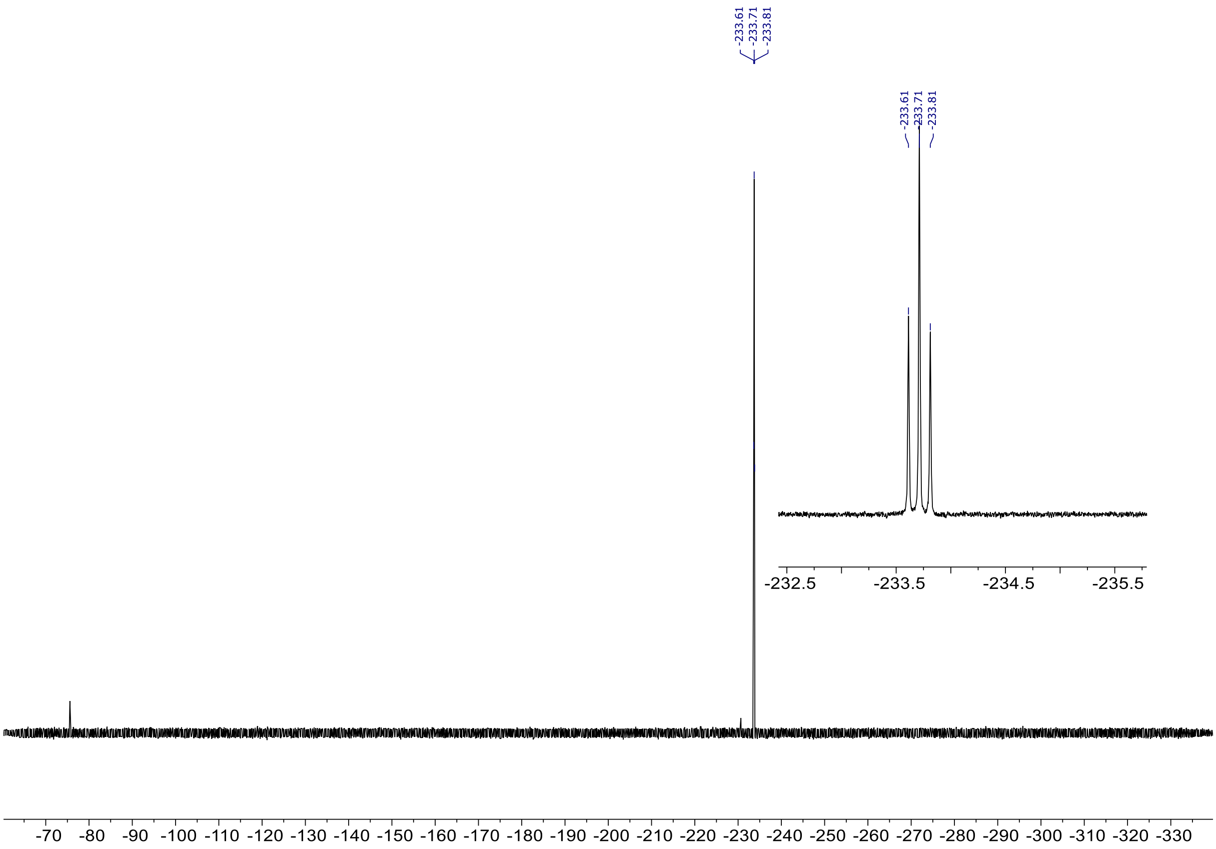

^{31}P-NMR spectrum of 16 (D_2O, 203 MHz)

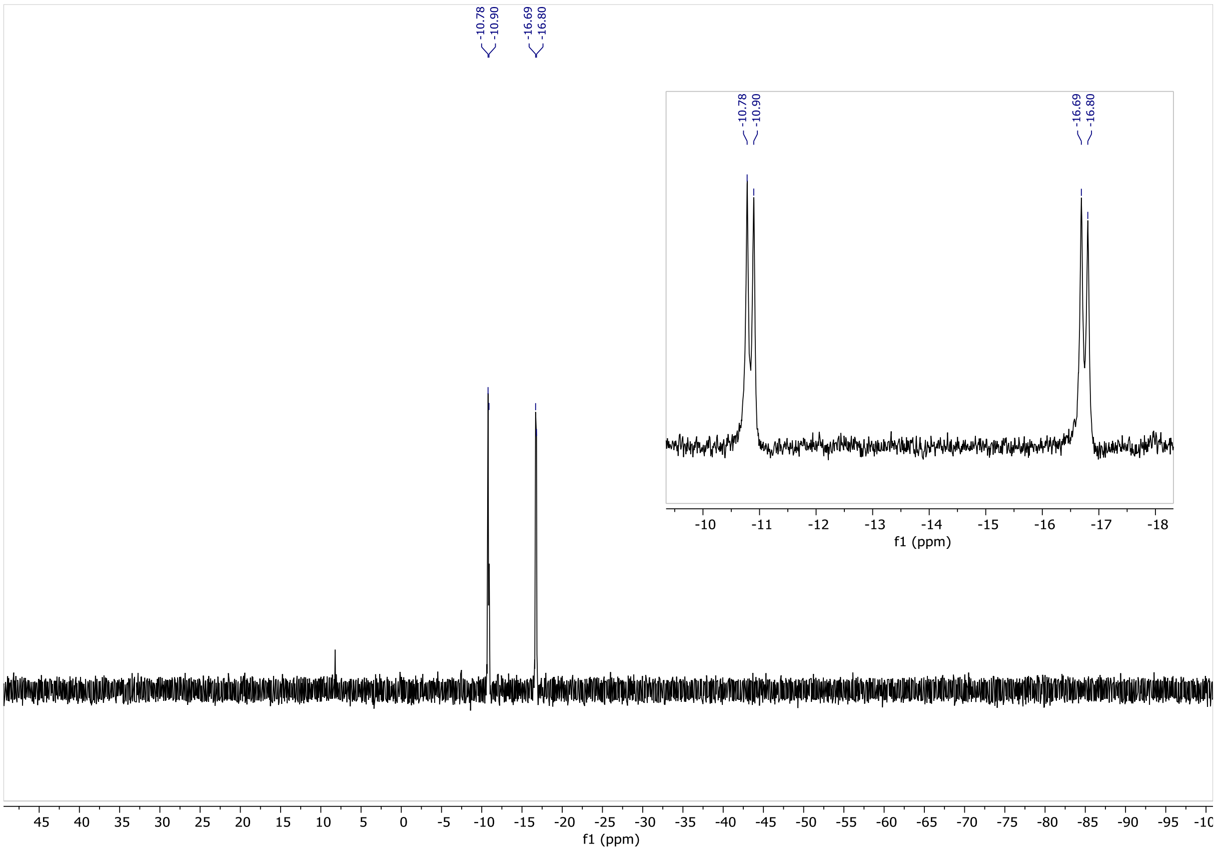

^{1}H-NMR spectrum of 8 (D_2O, 500 MHz)

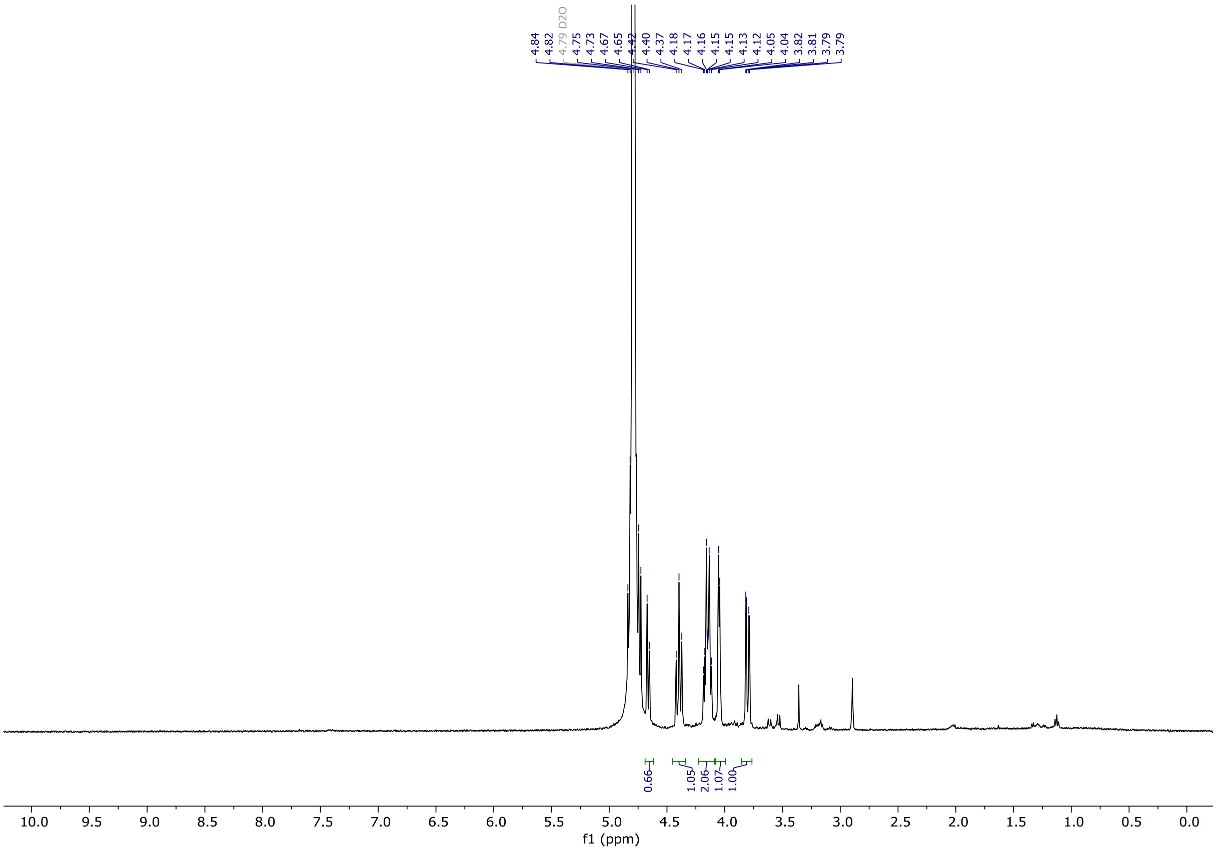

^{1}H-NMR spectrum of 8 (D_2O, 500 MHz) with presaturation of solvent signal at 4.78 ppm

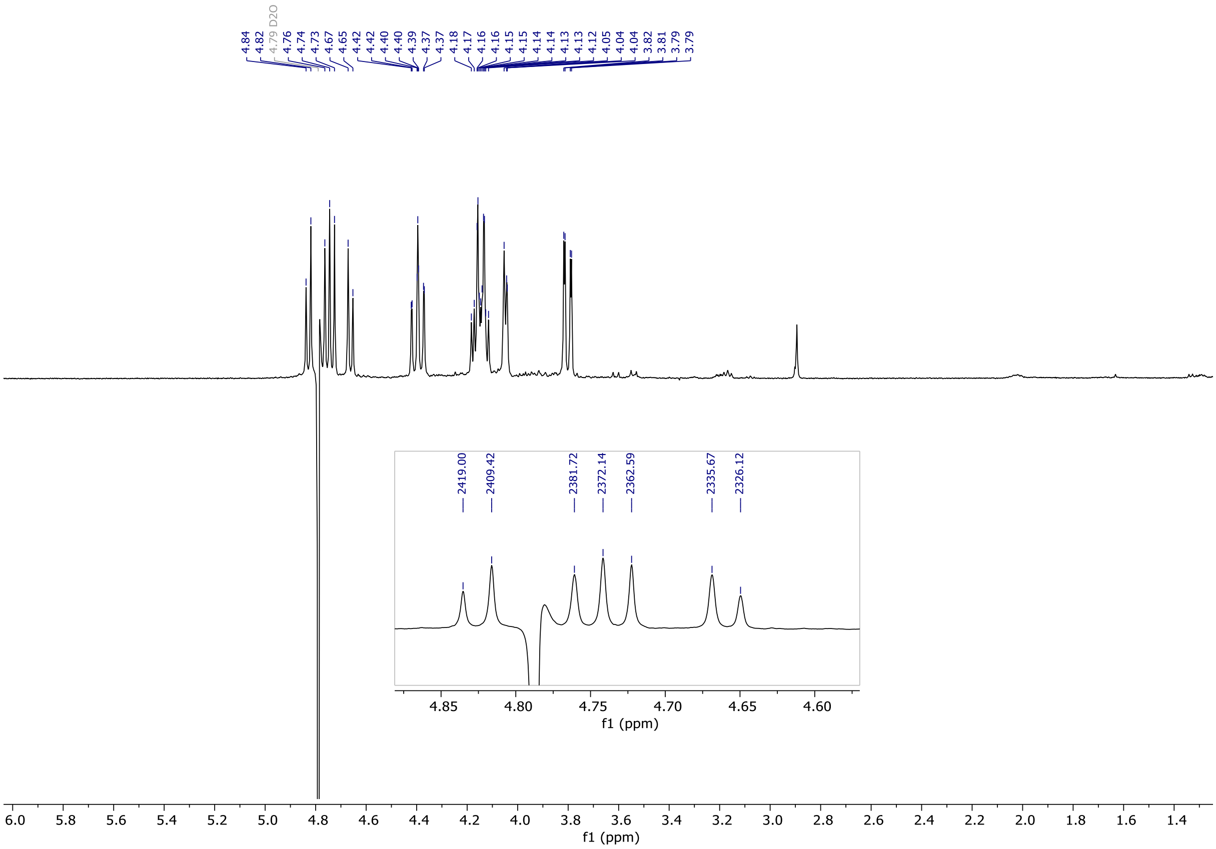

^{13}C-NMR spectrum of 8 (D_2O, 126 MHz)

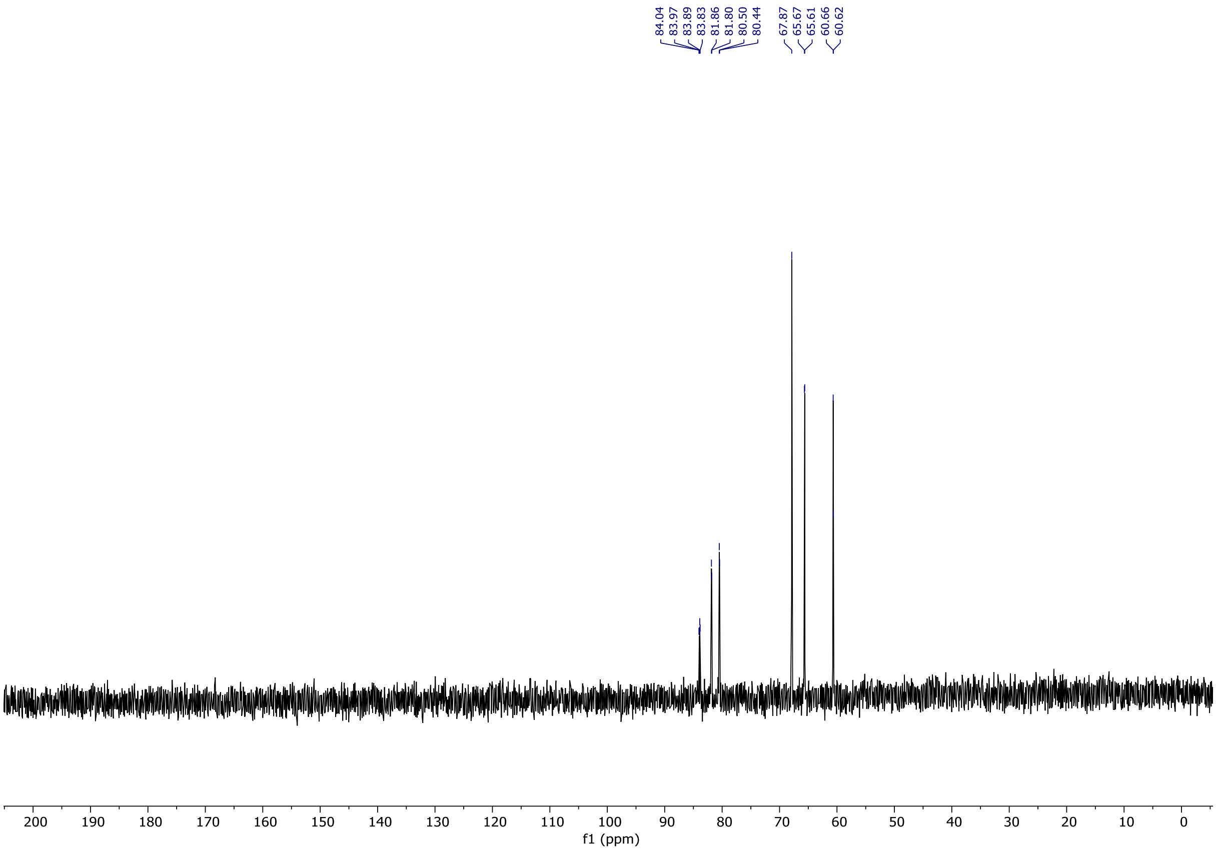

^{19}F-NMR spectrum of 8 (D_2O, 471 MHz)

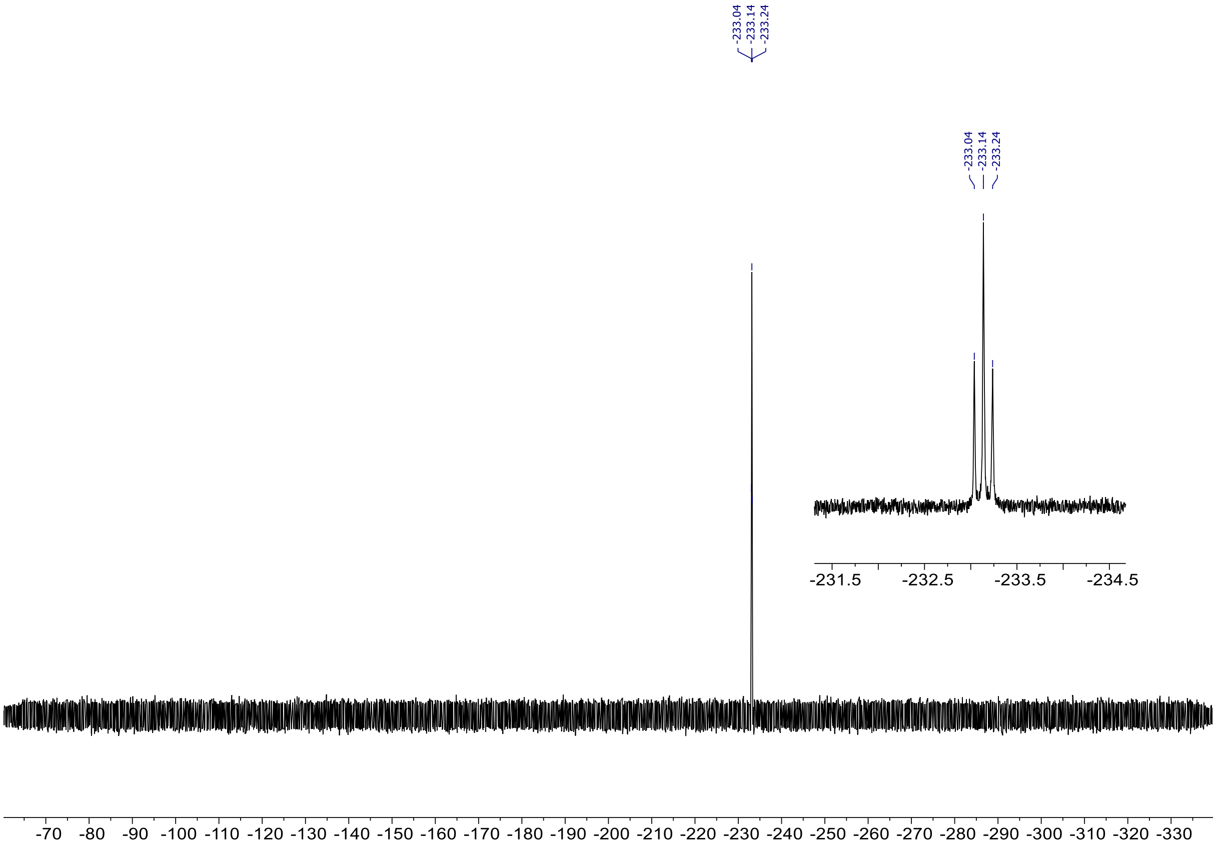

^{31}P-NMR spectrum of 8 (D_2O, 203 MHz)

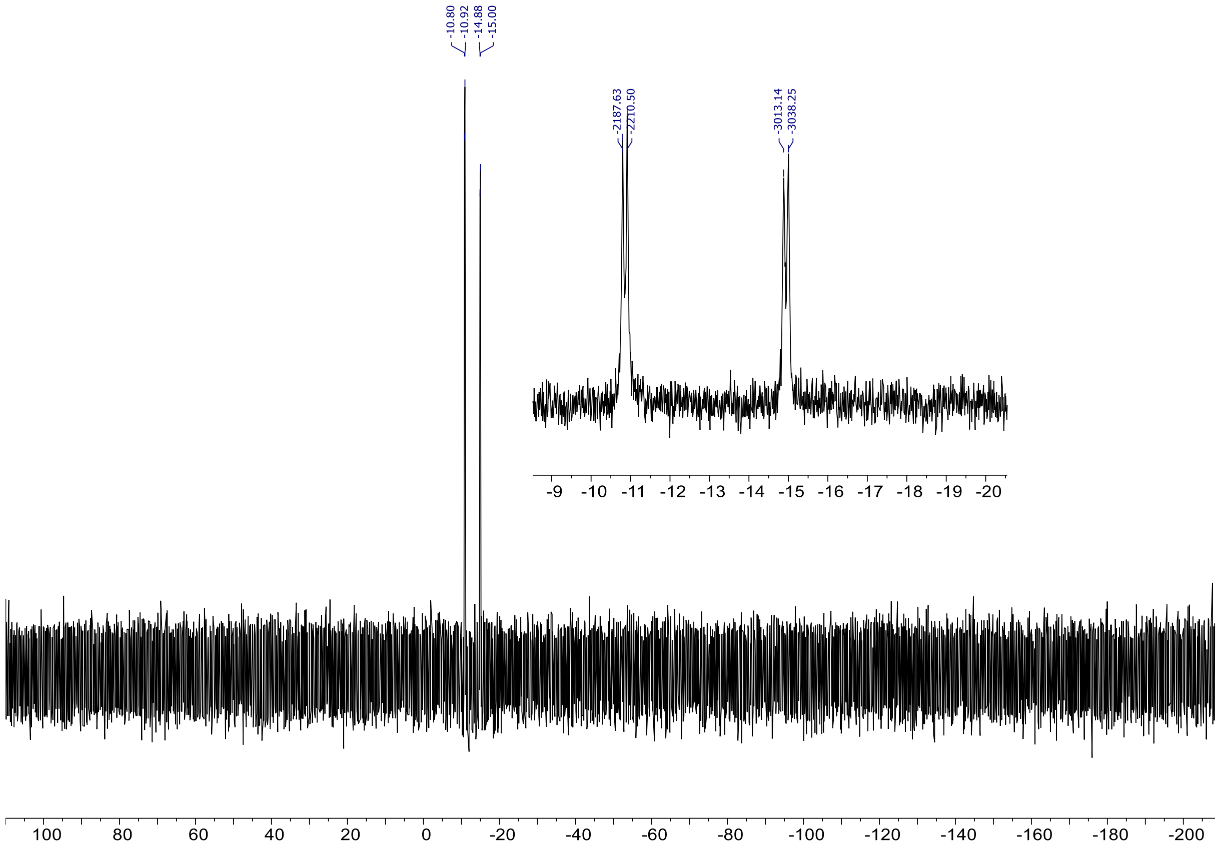

Biology

Biological procedures

***E. coli* IspG**. IspG was produced and purified in its dimeric form as previously reported.[4] Iron was quantified by the method of Fish[5] and sulphide by the method of Beinert[6] resulting in 3.3 iron and over 2.9 sulphur ions per IspG molecule.

Enzymatic assays of *E. coli* IspG. In a typical IspG assay (150 µL final volume), a solution of IspG (4 µM final concentration) was added using a gas-tight syringe to initiate the reaction in a 0.1 cm light path quartz cuvette containing NADPH (2 mM), flavodoxin (FldA, 60 µM), flavodoxin reductase (FpR1, 60 µM) and MEcPP (250 µM) in 50 mM Tris-HCl pH 8, previously prepared in an anaerobic glove box and pre-incubated at 37 °C for 3 min. The reaction was followed spectrophotometrically at 340 nm with a Cary 100 UV/visible spectrophotometer (Varian) kept at 37 °C using a thermostat equipped with a Peltier element. A control assay was performed by replacing IspG by buffer.

Inhibition assays: IspG (4 µM) was added via a gas-tight syringe to a 0.1 cm light path quartz cuvette prepared in an anaerobic glove box and containing NADPH (2 mM), FldA (30 µM), FpR1 (17 µM), MEcPP (250 µM) and **8** (250 µM) in 50 mM Tris-HCl buffer pH 8 using the classical protocol described above using UV/visible spectroscopy to monitor the reaction. The same assay was also performed by adding MEcPP to initiate the reaction using the same concentrations of MEcPP, IspG, **8** and cofactors as for the assay initiated by IspG.

IC_{50} determination: A MEcPP solution (final concentration 250 µM) was added through a gas-tight syringe to a 0.1 cm light path cuvette prepared in an anaerobic glove box and containing NADPH (2 mM), FldA (30 µM), FpR1 (17 µM), IspG (4 µM) and various concentrations of inhibitor in 50 mM Tris-HCl pH 8 that had previously been incubated for 15 min at 37 °C. The reaction was monitored as described above.

The initial velocity at each inhibitor concentration was plotted as dose-response curves and fit using Kaleidagraph according to the equation $y= 1/(1+ (x/IC_{50})^{slope})$ in order to determine the IC_{50} value with y representing the fraction of inhibition and x the inhibitor concentration.

IspG incubation using 8 as substrate: A 500 µL solution containing IspG (30 µM), NADPH (2 mM), flavodoxin (FldA, 60 µM), flavodoxin reductase (FpR1, 60 µM) and **8** (1 mM including *ca* 10 mol% of ammonium trifluoromethyl acetate) in 50 mM Tris-HCl pH 8 was incubated at 37 °C for 16 h. Subsequently, the solution was filtered on a centrifugal filter (Amicon Ultra 10K, Merck Millipore) that had previously been washed with 500 µL H_2O. The filtrate was recovered, removed from the glove box and lyophilized. The residue was dissolved in D_20 before ^1H and ^{19}F-NMR spectra were recorded.

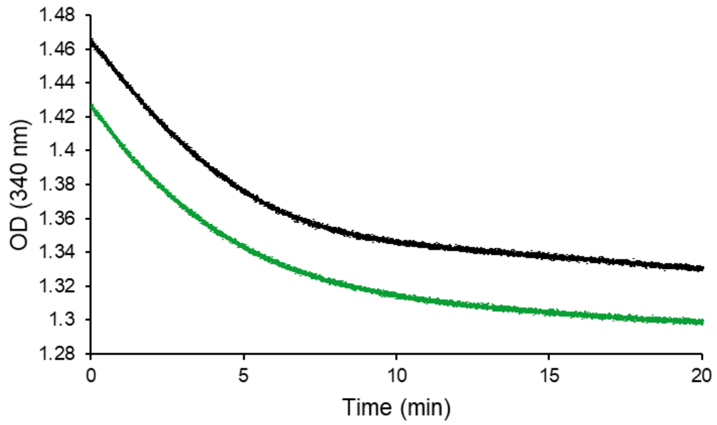

Figure S2: Decrease of the absorbance of NADPH at 340 nm in an IspG assay in which the reduction system was omitted during the pre-incubation of IspG with 8.

Conditions: NADPH (2 mM), FldA (60 µM), FpR1 (60 µM), GcpE (4 µM) and MEcPP (250 µM) in 50 mM Tris·HCl buffer pH 8 at 37 °C. IspG was pre-incubated in the presence of **8** 0 µM (black) and 250 µM (green) for 15 min at 37 °C before being added to the assay with a gas-tight syringe to initiate the reaction.

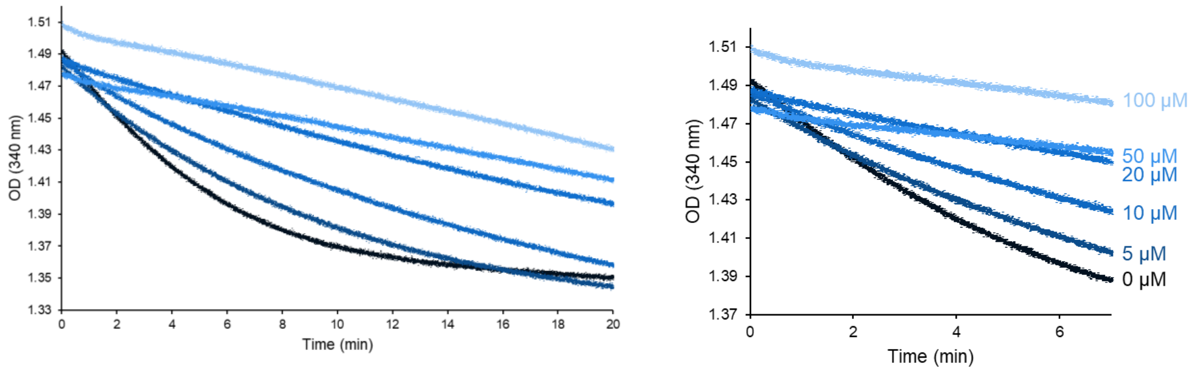

Figure S3: Decrease of the absorbance of NADPH at 340 nm with varying concentrations of 8 and 30 min pre-incubation time at 37 °C with IspG.

Conditions: NADPH (2 mM), FldA (60 µM), FpR1 (60 µM) and IspG (4 µM) in 50 mM Tris HCl buffer pH 8 and the following concentrations of **8**: 0 µM, 5 µM, 10 µM, 20 µM, 50 µM and 100 µM (from black to increasingly lighter shades of blue). The assays were initiated by the addition of MEcPP (250 µM) with a gas-tight syringe.

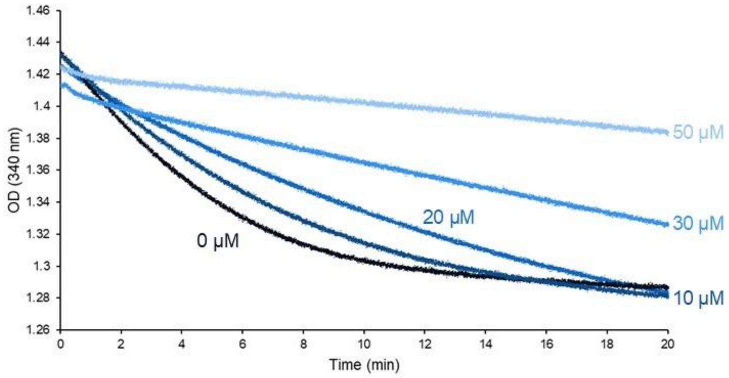

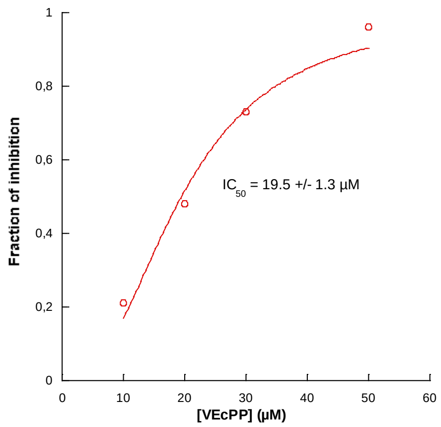

Figure S4: A. Decrease of the absorbance of NADPH at 340 nm with varying VEcPP concentrations and 30 min pre-incubation time at 37 °C; B. Fraction of inhibition of GcpE versus VEcPP concentrations deduced from A.

Conditions: NADPH (2 mM), FldA (60 µM), FpR1 (60 µM) and GcpE (4 µM) in 50 mM Tris HCl buffer pH 8 and the following concentrations of VEcPP: 0 µM (black), 10 µM, 20 µM, 30 µM and 50 µM (from dark to lighter shades of blue). The assays were initiated with the addition of MEcPP (250 µM) with a gas-tight syringe.

Control experiments to confirm that 8 targets IspG and not the reduction system. NADPH, FldA and FpR1 were pre-incubated with **8** for 30 min in absence of IspG, after which **8** was removed from the solution using a centrifugal filter. An IspG assay was then initiated using this reduction system and compared to an assay using a reduction system that was not exposed to **8**. The resulting progress curves were nearly identical (Figure S5), indicating no loss of IspG activity and confirming that **8** does not interfere with the reduction system.

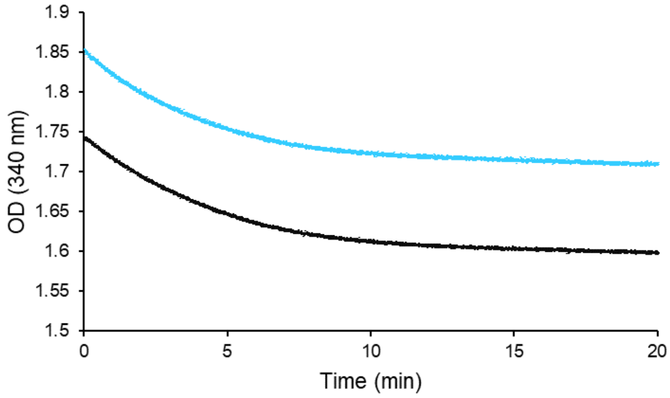

Figure S5: Decrease of the absorbance of NADPH at 340 nm in an IspG assay after pre-incubation of the reduction system with 8 and omitting IspG from the pre-incubation.

Conditions: NADPH (2.1 mM), FldA (60 µM), FpR1 (60 µM), IspG (4 µM) and MEcPP (250µM) in 50 mM Tris HCl buffer pH 8 preincubated in the presence of **8** (0 µM (black) and 1 mM (blue)) for 30 min at 37 °C before removal of **8** by centrifugation-dilution cycles. IspG was added with a gas-tight syringe to initiate the reaction.

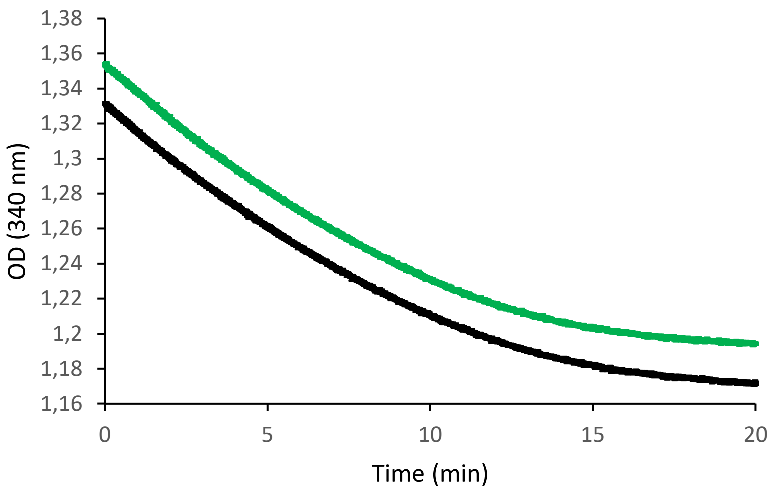

Figure S6: Decrease of the absorbance of NADPH at 340 nm in an IspG assay without (black) and with TFA (green).

Conditions: NADPH (2 mM), FldA (30 µM), FpR1 (17 µM), IspG (4 µM) and MEcPP (250 µM) in 50 mM Tris·HCl buffer pH 8 at 37 °C. The reduction system and MEcPP were pre-incubated for 3 min at 37 °C before IspG was added to the assay with a gas-tight syringe to initiate the reaction (black). IspG and the reduction system were pre-incubated in the presence of 250 µM TFA for 15 min at 37 °C before MEcPP was added to the assay with a gas-tight syringe to initiate the reaction (green).

^{1}H and ^{19}F-NMR spectra of enzymatic mixtures

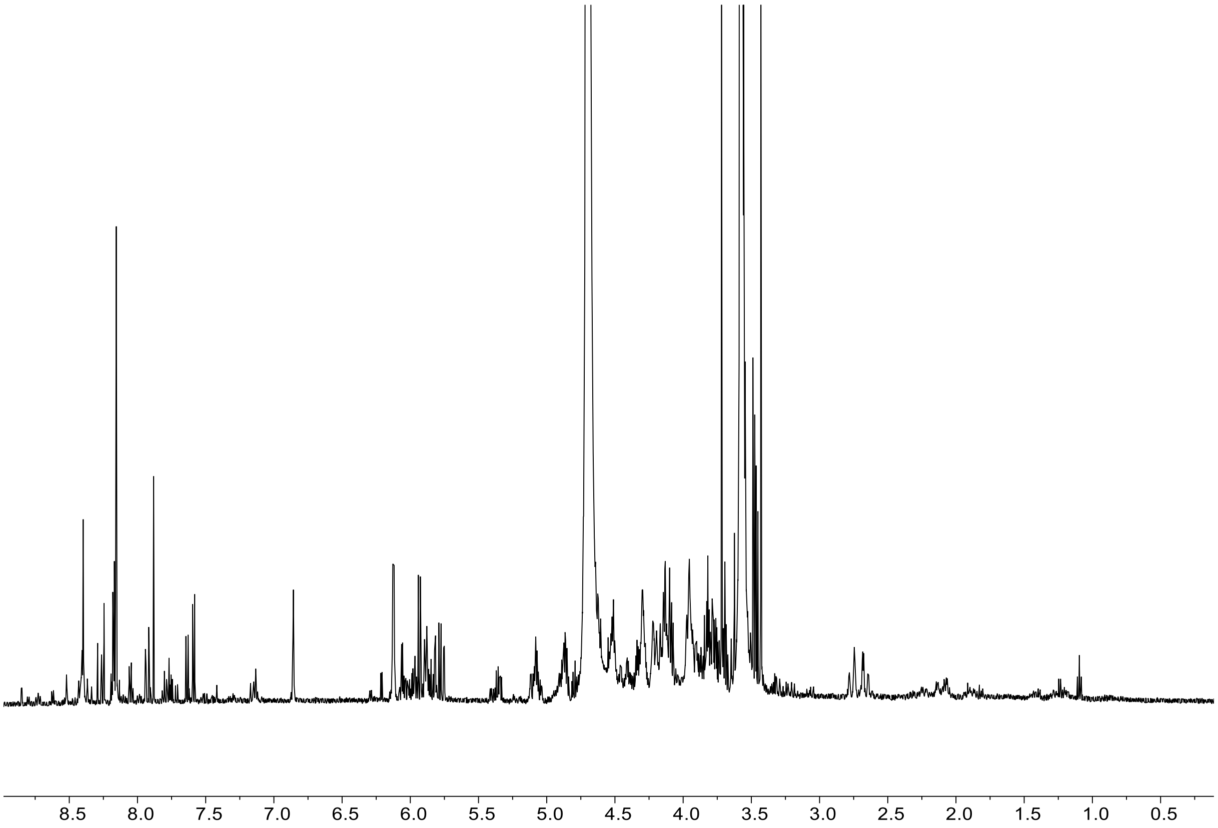

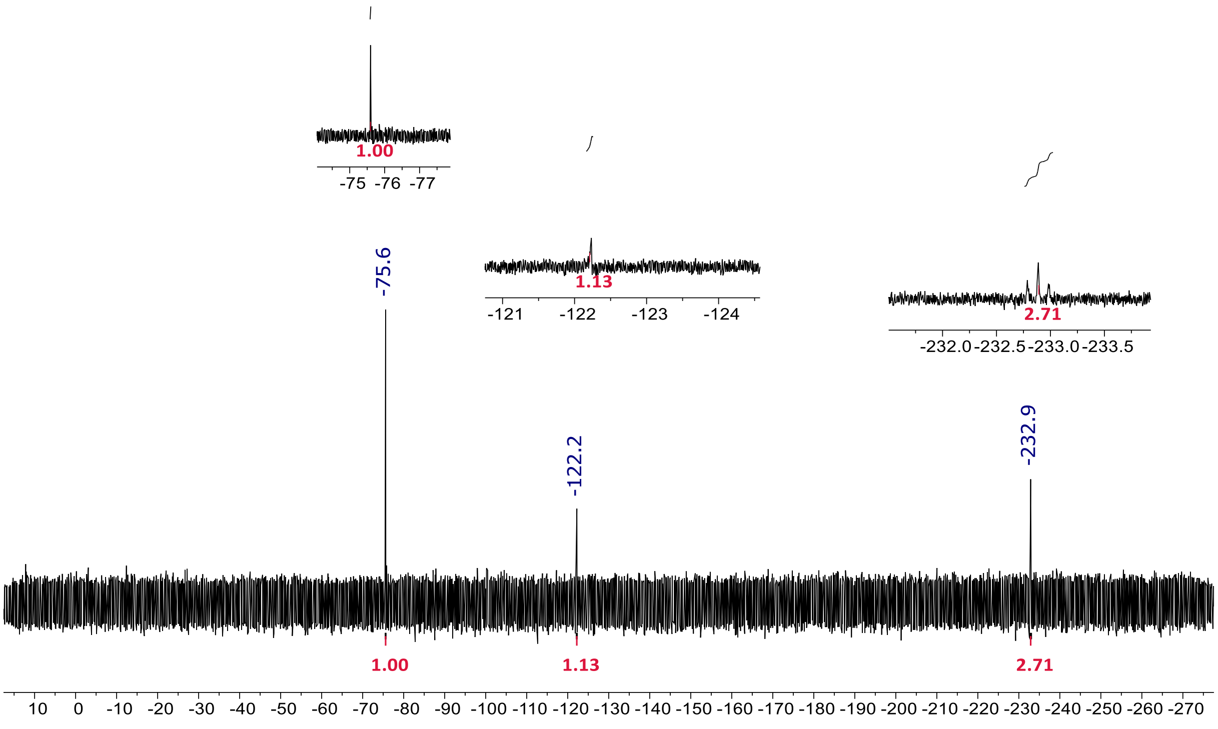

References

[1] Patrick J. Hrdlicka, Nicolai K. Andersen, Jan S. Jepsen, Flemming G. Hansen, Kim F. Haselmann, Claus Nielsen and Jesper Wengel, Synthesis and biological evaluation of branched and conformationally restricted analogs of the anticancer compounds 3'-C-ethynyluridine (EUrd) and 3'-C-ethynylcytidine (ECyd), Bioorg. & Med. Chem. **2005**, *13*, 2597–2621.

[2] Joy Krishna Maity, Ramprasad Ghosh, Michael G. B. Drew, Basudeb Achari, Sukhendu B. Mandal, Introduction of Vinyl and Hydroxymethyl Functionalities at C-4 of Glucose-Derived Substrates: Synthesis of Spirocyclic, Bicyclic, and Tricyclic Nucleosides, *J. Org. Chem.* **2008**, *73*, 4305–4308.

[3] Subhankar Tripathi, Biswajit G. Roy, Michael G. B. Drew, Basudeb Achari, Sukhendu B. Mandal, Synthesis of Oxepane Ring Containing Monocyclic, Conformationally Restricted Bicyclic and Spirocyclic Nucleosides from D-Glucose: A Cycloaddition Approach, *J. Org. Chem*. **2007**, *72*, 7427-7430.

[4] Basile Simonet, Vivien Herrscher, Clea Witjaksono, Philippe Chaignon, Fabien Massicot, Jean-Luc Vasse, Myriam Seemann, Jean-Bernard Behr, Carbohydrate-Templated Syntheses of Trifluoromethyl-Substituted MEP Analogues for the Study of the Methylerythritol Phosphate Pathway, *J. Org. Chem.* **2023***, 88, 15832-15843.*

[5] Wayne W. Fish, Rapid colorimetric micromethod for the quantitation of complexed iron in biological samples, *Methods Enzymol.* **1988**, *158*, 357-364.

[6] Helmut Beinert, Semi-micro methods for analysis of labile sulfide and of labile sulfide plus sulfane sulfur in unusually stable iron-sulfur proteins, *Anal. Biochem.* **1983**, *131*, 373-378.
